# Immunometabolism and Pulmonary Infections: Implications for Protective Immune Responses and Host-Directed Therapies

**DOI:** 10.3389/fmicb.2019.00962

**Published:** 2019-05-07

**Authors:** Martin Rao, Ernest Dodoo, Alimuddin Zumla, Markus Maeurer

**Affiliations:** ^1^ImmunoSurgery Unit, Champalimaud Centre for the Unknown, Lisbon, Portugal; ^2^Department of Oncology and Haematology, Krankenhaus Nordwest, Frankfurt, Germany; ^3^Division of Infection and Immunity, University College London, NIHR Biomedical Research Centre, University College London Hospitals NHS Foundation Trust, London, United Kingdom

**Keywords:** lung infections, immunometabolism, inflammation, immunological memory, protective immune responses

## Abstract

The biology and clinical efficacy of immune cells from patients with infectious diseases or cancer are associated with metabolic programming. Host immune- and stromal-cell genetic and epigenetic signatures in response to the invading pathogen shape disease pathophysiology and disease outcomes. Directly linked to the immunometabolic axis is the role of the host microbiome, which is also discussed here in the context of productive immune responses to lung infections. We also present host-directed therapies (HDT) as a clinically viable strategy to refocus dysregulated immunometabolism in patients with infectious diseases, which requires validation in early phase clinical trials as adjuncts to conventional antimicrobial therapy. These efforts are expected to be continuously supported by newly generated basic and translational research data to gain a better understanding of disease pathology while devising new molecularly defined platforms and therapeutic options to improve the treatment of patients with pulmonary infections, particularly in relation to multidrug-resistant pathogens.

## Background

Central immune effector functions, e.g., the development of long-term immunological memory, homing to target tissues and effective immune-surveillance are, in part, determined by metabolic programming, which plays a role not only in cellular physiology yet also in immunopathology (Shehata et al., 2017; Gardiner, 2019). A better understanding of the dynamic role of metabolic programming may devise new ways of targeted therapeutic intervention(s). Preclinical and clinical studies in patients with non-communicable diseases link metabolic cellular impairment with immune dysfunction (Hotamisligil, 2017). A shift in metabolite requirement appears to govern the nature and dynamics of the immune response in the host – largely involving glucose or lipids and fatty acids (FA) (Lochner et al., 2015; Wei et al., 2017). For example, aberrant glucose metabolism and subsequent impaired immune function in patients with diabetes mellitus (DM) increases the risk for infections that require hospitalization (Shah and Hux, 2003). Dietary practices, i.e., Mediterranean eating habits versus a classical, high-fat Western diet underline how a ‘healthy diet’ is generally able to reduce inflammation and infections (Martínez-González et al., 2017). High blood glucose levels are associated with a higher incidence of pulmonary tuberculosis (TB) – predisposing patients with type 2 DM (T2DM) to a higher risk of TB-related mortality despite antimycobacterial therapy (Faurholt-Jepsen et al., 2013). The World Health Organisation (WHO) and global clinical TB working groups have identified DM to be a major comorbidity for TB susceptibility (Marais et al., 2013; Lonnroth et al., 2014; Girardi et al., 2017). We review here the role of immunometabolism in the tissue microenvironment, the axis of the intestinal microbiome and tissue-associated immune responses and discuss how targeted therapies may shift the balance of dysfunctional, damaging or non-productive immune responses to protective immune reactivity patterns that allow the containment of pathogens – or elimination of transformed host cells.

## The Diseased Tissue Microenvironment as a Critical Modulator of Immunometabolism

In T cells, glucose metabolism via the glycolytic pathway as opposed to fatty acid oxidation (FAO) differs between various T-cell maturation/differentiation subtypes, i.e., those expressing the ‘effector,’ ‘memory,’ and the ‘exhausted’ functional phenotypes. The transmembrane channel glucose transporter 1 (Glut1) is mandatory for cellular glucose uptake to fuel mitochondrial ATP generation, which plays a cardinal role in initiating IFN-γ gene transcription during an inflammatory response to fuel anti-tumor T-cell functions and cellular proliferation (Buck et al., 2017; Wei et al., 2017). Glut1 is induced by interleukin (IL) 7, a quintessential homeostatic cytokine necessary for the formation and maintenance of memory T-cell responses (Wofford et al., 2008). Along with the engagement of signal transducer and activator of transcription 5 (STAT5), IL-7 promotes cellular glucose uptake and survival (Wofford et al., 2008). The biological significance of IL-7 has been consolidated in translational and clinical studies to improve host immunity to infectious diseases by activation of CD4+ T-cell populations and re-programming of the antigen-specific T-cell repertoire in the context of TB, human immunodeficiency virus (HIV) infection and sepsis in preclinical (Maeurer et al., 2000; Vassena et al., 2012; Rao et al., 2013; Kulkarni et al., 2018) as well as clinical settings (Sportes et al., 2008; Levy et al., 2009; Francois et al., 2018).

The presence of extracellular lactate has been shown to be associated with (human) T-cell proliferation *in vitro* and CD4+ T-cell activity (Grist et al., 2018). Importantly, CD4+ effector T cells also produce lactate which abrogates regulatory T-cell (Treg) responses and promotes Th17 development (Haas et al., 2015; Grist et al., 2018), which is reversible by blocking aerobic glycolysis (Haas et al., 2015; Eleftheriadis et al., 2016). However, an earlier study showed that lactate produced by tumor cells can inhibit cytolytic activity of human CD8+ effector T cells *in vitro* (Fischer et al., 2007). Memory CD8+ T cells rely more heavily on fatty acid oxidation (FAO) compared to effector T cells, where glucose breakdown leading to pyruvate production is crucial (Pearce et al., 2009; O’Sullivan et al., 2014). Tregs also rely greatly on FA metabolism in an adenosine monophosphate-activated protein kinase (AMPK)-dependent manner, therefore raising the possibility of Treg survival in an environment enriched with high bioavailability of FA species (Newton et al., 2016).

*Mycobacterium tuberculosis* (*M.tb*)-infected macrophages experience a shift from oxidative phosphorylation (OXPHOS) to aerobic glycolysis *in vitro*, which leads to abrogated IL-1β production and increased IL-10 synthesis associated with intracellular mycobacterial growth (Gleeson et al., 2016). This also resonates with the observation that individuals with T2DM, having high blood glucose, exhibit impaired TB disease control (Wang et al., 2009; Ferrara et al., 2012; Faurholt-Jepsen et al., 2013). In mice infected with *M.tb*, Glut1 transcription is upregulated, in addition to the transporter associated with lactate secretion (Shi et al., 2016), which might have a negative effect on bystander effector CD8+ T cells (Shehata et al., 2017). Of note, glucose transporters (including Glut1) as well as the ADP-dependent glucose kinase (ADPGK), which is associated with metabolic T-cell activation in active pulmonary TB, are also upregulated in lung granulomas from patients with active TB (Subbian et al., 2015; Shi et al., 2016). The lung pathogens *Staphylococcus aureus* and *Bordetella pertussis* also perpetrate dysregulated glucose metabolism in the host, with the latter directly causing insulin resistance by negatively regulating blood glucose homeostasis (Vitko et al., 2015; Bischoff et al., 2017; Freyberg and Harvill, 2017). Rats fed with a high-fat diet (in relation to obesity) were shown to present with an accumulation of inflammatory macrophages characterized by Glut1 upregulation as well as IL-6 and TNF-α expression in adipose tissue and the liver (Freemerman et al., 2014). Glut1 overexpression enhanced glucose uptake and glycolysis in these macrophages, further to upregulation of other pro-inflammatory mediators such as CCL5 (also called RANTES), necessary for CD8+ T-cell activity against viral infections (Crawford et al., 2011) and granulocyte-colony-stimulation factor (G-CSF), which promotes neutrophil growth, downregulation of IL-17 production (Martins et al., 2010) and potentially expands central memory G-CSF receptor-expressing CD4+ IL-4+ Th2 cells in human blood (Malashchenko et al., 2018). Immunological mediators, measured at various time points in individuals with metabolic disorders, i.e., obesity and diabetes, may hold great clinical value in terms of preventing full-fledged pulmonary infections particularly TB with respect to devising host-directed immunotherapeutic interventions (Rao et al., 2019a,b).

Disbalance in glucose metabolism triggered by influenza virus has been reported in pediatric patients, which was found to be reversible by pharmacological inhibition of the phosphatidylinositol-3-kinase/mammalian target of rapamycin (PI3K/mTOR) pathway (Smallwood et al., 2017). Pertaining to HIV infection of macrophages, the glycolysis-associated enzyme hexokinase 1 (HK-1) has been shown to bind to mitochondria to increase its membrane potential and support the survival and maintenance of infected cells. The common antifungal agent clotrimazole can inhibit HK-1 activity in macrophages, thereby unleashing caspase 3/7-mediated apoptosis (Sen et al., 2015). Inhibition of HK-2 can has also been shown to promote skewing of human CD4+ T cells to acquire a regulatory phenotype (Eleftheriadis et al., 2016). Enhanced mitochondrial membrane potential to support pathogen replication has also been attributed to the infection of epithelial cells with *Chlamydia pneumoniae*, an important intracellular bacterial parasite, under normoxic conditions further to increased expression of NADPH in host cells (Käding et al., 2017). Glucose uptake is also necessary for B-cell proliferation and antibody production, which has been shown to depend on PI3K activity in association with an increase in lactate production (Doughty et al., 2006; Caro-Maldonado et al., 2014). This balance is disrupted in patients with T2DM, manifesting in exaggerated and deleterious pro-inflammatory immune responses (Deng et al., 2017) which possibly makes patients with T2DM more susceptibility to TB and other pulmonary infections compared to non-diabetic individuals (Casqueiro et al., 2012; Rao et al., 2015). Collectively, these ‘shunting’ mechanisms of host metabolism support pathogen survival either directly or by manipulating the immune system and may, therefore, represent an ‘immune escape strategy’ used by pathogens.

New research has shed light on the role of glycolysis in subduing T-cell infiltration into tumors as well as the ability of T cells to kill target cells, concomitant with downregulation of interferon regulator factor 1 (IRF1), an essential interferon gamma (IFN-γ) response element (Cascone et al., 2018). Since host molecules linked to glucose transport and metabolism are activated in human TB granulomas, it appears that the glycolytic pathway could potentially affect effective T-cell responses in pulmonary TB. Glucose metabolism in CD4+ T cells during chronic HIV infection also promotes activation, marked by Glut1 and upregulation of the major histocompatibility class II (MHC-II) molecule human leukocyte antigen-DR (HLA-DR) (Palmer et al., 2014b), a marker of T-cell activation. At the same time, this T-cell subset is also depleted regardless of antiretroviral therapy (ART). A similar pattern of increased glucose metabolism and Glut1 expression profile has been observed in monocytes derived from patients with HIV, irrespective of ART status (Palmer et al., 2014a).

L-arginine is also necessary for T-cell survival; elevated levels are metabolized by arginase 2 (ARG2) in mitochondria to induce OXPHOS, switching from glycolysis, for production of ATP via the electron transport chain (Geiger et al., 2016). Increased L-arginine uptake by T cells promotes the expansion of central memory T-cell subsets (CCR7+) in addition to acquisition of effector functions, i.e., IFN-γ production even in the absence of T-cell homeostatic cytokines such as IL-2 or IL-15 (Geiger et al., 2016). *M.tb*-infected macrophages present at the hypoxic center in TB lung granulomas in a murine model of pulmonary TB have been previously shown to utilize ARG1, *in lieu* of NOS2, to catabolize L-arginine (Duque-Correa et al., 2014). This reduces T-cell proliferation and the resulting immunopathology while abrogation of ARG1 enzymatic activity exacerbates lung pathology (Duque-Correa et al., 2014).

Excessive glucose uptake by activated T cells as well as macrophages during inflammation has been observed in conjunction with hypoxia. Response to hypoxia by ‘foamy’ macrophages in atherosclerotic plaques as well as migratory CD8+ T cells during inflammation, marked by hypoxia-inducible factor 1 alpha (HIF-1a) expression, has been observed to elevate glucose uptake (Folco et al., 2011; Finlay et al., 2012). ‘Foamy’ macrophages are cytoplasmic lipid-enriched cells associated with atherosclerotic plaques which, due to dysregulation of cholesterol metabolism, accumulate intracellular cholesteryl ester deposits (Moore et al., 2013). Hypoxic TB lesions/granulomas in the lung have also been shown to display an accumulation of ‘foamy’ macrophages which can be induced by *M.tb*-derived lipids, i.e., mycolic acids species (Peyron et al., 2008) as well as host-derived triglyceride esters in necrotic lesions (Guerrini et al., 2018; Jaisinghani et al., 2018) and are capable of local modulation of T-cell responses (Prosser et al., 2017). Although yet to be formally tested, hypoxia-induced ‘foamy’ macrophage accumulation may also pose the likelihood of affecting positron emission tomography-computed tomography (PET-CT) readouts in pulmonary TB imaging due to the increased glucose uptake by and glycolytic activity of macrophages in granulomas (Fu et al., 2004; Singh et al., 2015; Geadas et al., 2018). In addition, recent evidence suggests that *M.tb*-infected macrophages accumulate intracellular lipid droplets following IFN-γ-dependent immune activation rather by direct, pathogen-derived mechanisms (Knight et al., 2018). This feature was found to benefit the host in a murine model of pulmonary TB, although the metabolic profile of immune cells is likely to shift with a high amount of lipid intake – potentially promoting FAO and longevity of the cells in tissue (Remmerie and Scott, 2018).

The afore-mentioned HIF-1α has also been shown to be induced in HIV-infected macrophages, involved in the upregulation of HK-1 for supporting viral replication and biogenesis (Barrero et al., 2013), warranting further elucidation of the HIF-1α axis in TB/HIV co-infection in relation to immunometabolism. *C. pneumoniae*-infected epithelial cells have also been shown to upregulate enzymes involved in hypoxia-induced glycolysis, i.e., phosphofructokinase, lactate dehydrogenase and glycerol-3-phosphate dehydrogenase which promote pathogen survival (Szaszak et al., 2013). Furthermore, *C. pneumoniae* infection itself directly stabilizes HIF-1α expression to enhance glycolysis (Rupp et al., 2007), which is also likely to dampen HLA class I antigen processing, presentation and immune surveillance as observed in cancer (Sethumadhavan et al., 2017). Epstein-Barr virus (EBV) and CMV are known downmodulators of the HLA class I pathway (Levitskaya et al., 1995; Benz et al., 2001), while *M.tb* early-secreted antigenic target of 6 kDA (ESAT-6), an immunodominant antigen, has been shown to interfere with beta-2-microglobulin (β2M) insertion into the HLA class I complex within the endoplasmic reticulum (Sreejit et al., 2014). Thus, testing whether alteration of glucose uptake during infection with intracellular pathogens would improve HLA class I antigen presentation using clinically relevant models would be very useful to devise immuno-stimulatory interventional strategies.

### Fatty Acids and Immunometabolism

The availability of free fatty acids (FFA) and the intracellular biosynthesis of lipid droplets in T cells following glucose metabolism has a drastic impact on T-cell functionality and polarization (de Jong et al., 2014). As mentioned previously, FAO is a quintessential metabolic program in memory CD8+ T cells for their development, sustenance and immuno-protective activity in the host (Pearce et al., 2009; van der Windt et al., 2012; O’Sullivan et al., 2014). Saturated FFA, i.e., palmitic acid, lauric acid, myristic acid as opposed to unsaturated FA, such as oleic acid and linoleic acid, appear to be generally less toxic and promote *less* inflammatory responses by human T cells (Lima et al., 2002; Cury-Boaventura et al., 2004, 2006). Conversely, saturated FA have been shown to impair HLA class I antigen processing and presentation, resulting in reduced recognition and killing of target cells by FA-exposed human CD8+ T cells (Shaikh et al., 2008). Similarly, palmitic acid-treated APCs were also unable to activate naïve CD8+ T cells, which resonates with the earlier finding that saturated FA do not trigger strong inflammatory responses (Shaikh et al., 2008). In addition to CD8+ T-cell activation, a reduced exogenous supply of FA has also been attributed to compromised HLA-DR (major HLA class II molecule in humans) expression profile, in part owing to impaired lysosomal activity – which was rescuable with coenzyme-A (CoA)-coupled unsaturated FA, i.e., oleic and linoleic acids (Schweitzer et al., 2006). Human adipose tissue-derived stem cells (ASCs) from obese individuals have also been shown to exhibit increased HLA-DR expression albeit with lesser cytoplasmic lipid-droplet accumulation (Pachón-Peña et al., 2016), possibly due to increased cellular oxygen consumption leading to depletion of fat deposits in ASCs (Perez et al., 2015). Another preclinical study in mice provided evidence that a high fat diet-induced upregulation of MHC-II, leading to generally increased inflammatory responses, lowered anti-inflammatory responses and M1-polarized pro-inflammatory macrophages (Deng et al., 2013). While in the afore-mentioned study this MHC-II-driven immune activation was identified as an important perpetrator of obesity, this may, on the contrary, be beneficial in the context of pulmonary infections at the initiation of host immune responses targeting pathogens (Zumla et al., 2016). Nutrition may, therefore, indirectly affect susceptibility to pulmonary infections – as with TB (Fox et al., 2015) and recurrent respiratory infections among children (Zhang et al., 2015). In addition to T cells, the requirement of FA by antigen-presenting cells (APCs), i.e., macrophages, dendritic cells and B cells has also been reported to be crucial for activation, phagosome–lysosome fusion, antigen processing and presentation via HLA-DR expression – as well as anchoring proteins into lipid rafts on the cell surface (Bouillon et al., 2003; Schweitzer et al., 2006).

The discussion presented above warrant deeper insights into the various mechanisms by which FA-mediated metabolic programming in immune cells, adaptive and innate alike, shape the immune repertoire in humans – particularly in pulmonary infections and associated diseases (Krishnamoorthy et al., 2018).

## Pulmonary Surfactants in the Context of Lung Immunometabolism

An important lipoprotein class unique to the lungs are pulmonary surfactants, which are produced by type II pneumocytes and crucial for reducing surface tension, gas exchange functions, tissue integrity and host defense (Wright, 2004). Two members of lung surfactants, namely SPA-A and SPD-D, also possess immune-related properties. SP-D is mainly associated with opsonization of Gram-negative bacteria by binding to the LPS moieties on the bacterial surface for subsequent engulfment by innate immune cells – which leads to activation of APCs and T-cell recruitment (Wright, 2004). SP-A, on the contrary, while capable of inducing IL-8 production by neutrophils and macrophage activation, can inhibit T-cell proliferation and the maturation of dendritic cells (Wright, 2004). While important in clearing Gram-negative bacterial infections, e.g., *Pseudomonas* sp. and streptococcal infections, individuals with chronic autoimmune manifestations, i.e., ARDS, sarcoidosis and pulmonary fibrosis also exhibit impaired surfactant functions (Hermans and Bernard, 1999). The homeostasis of cholesterol, the major neutral lipid of pulmonary surfactants, requires ATP-binding cassette transporter G1 (ABCG1) and ATP-binding cassette transporter A1 (ABCA1) activity without which cholesterol build-up in the alveolar air space can occur in addition to the accumulation of ‘foamy macrophages’ (Baldan et al., 2008; Wojcik et al., 2008; Draper et al., 2010). In keeping with this, ABCG1-deficient macrophages can also be found in patients with pulmonary alveolar proteinosis (PAP) – a rare disease characterized by the build-up of surfactant in the alveolar space (Borie et al., 2011) – in conjunction with autoantibodies against GM-CSF blocking its signaling cascade (Thomassen et al., 2007).

Impaired lipid efflux functions have been demonstrated in immune cells from patients with chronic lung diseases. Some examples are ABCA1 and ABCG1, downregulated in lung-resident immune cells from patients with sarcoidosis (Barna et al., 2016); compromised activity of the transcription factor liver X receptor (LXR); reduced plasma levels of alveoli-derived cholestenoic acid in patients with TB, emphysema as well as sarcoidosis (Babiker et al., 1999) and the absence of ABCG1 expression in PAP (de Aguiar Vallim et al., 2017). Accumulation of foamy cells in smokers – a strong comorbidity factor in lung infections (Wilson et al., 2011) – reflects impaired lipid cellular metabolism, as reduced levels of apolipoprotein A1 (ApoA1) are found in the bronchoalveolar lavage fluid of patients with idiopathic pulmonary fibrosis (IPF) (Kim et al., 2010). These indicators may provide a clinically relevant matrix concerning an individual’s susceptibility to contract pulmonary infection(s) (Glasser and Mallampalli, 2012) while aiding the development of targeted treatment strategies.

While patients with PAP harboring ATP-binding cassette deficiency are prone to opportunistic lung infections (Goldschmidt et al., 2003; Borie et al., 2011), TB or mycobacterial infections in general, it has also been implicated in the pathogenesis of sarcoidosis (van Enschot and van Balkom, 2013; Ferrara et al., 2017), suggesting an overall impaired host defense involving pulmonary surfactant disbalance. Interestingly, ABCA1 loss in murine alveolar macrophages enhanced pro-inflammatory immune responses in the lung and clearance of bacterial infection (Draper et al., 2010), which requires well-designed studies in humans. Taken together, surfactant/lipid homeostasis in the lung, pertaining to cholesterol metabolism, suggests a critical role in balancing pulmonary inflammation. Herein, the ABC transporters associated with lipid homeostasis in the lung play a crucial role in mediating local immune responses and pathogen control.

## Metabolic Reprogramming of Natural Killer Cells in Infected Tissue Compartments

Akin to metabolic changes experienced by T and B cells during pulmonary infections, natural killer (NK) cells also appear to undergo similar tissue-associated ‘metabolic stress’ which affects their capacity to clear infectious pathogens (Gardiner, 2019). While NK cells play a role in halting development of active TB in individuals with LTBI (Garand et al., 2018; Roy Chowdhury et al., 2018), circulating IL-6Rα+ NK cells in T2DM have been shown to perpetrate deleterious inflammation (Theurich et al., 2017), with the likelihood of affecting NK-cell activity in the lung. The functionality of NK cells in obese individuals also appears to be compromised and is further accentuated by exposure to cigarette smoke (O’Shea et al., 2010; Laue et al., 2015) – which subdues host defense mechanisms in general, particularly in the lungs (Bagaitkar et al., 2008; Lugade et al., 2014). In humans, CD56^bright^ NK cells exhibit reduced cytotoxic functions (granzyme, perforin production) albeit superior IFN-γ production compared to CD56^dim^ NK cells. Furthermore, CD56^bright^ NK cells constitute the most frequent tissue-resident population, while CD56^dim^ NK cells comprise approximately 90% of circulating NK cells (Gardiner and Finlay, 2017). Adaptive, memory-like NK cells have also been described to share similarities with memory CD8+ T cells in terms of DNA promoter region hypermethylation patterns (Schlums et al., 2015). Memory-like NK cells display reduced expression of the Fc gamma receptor III (FcγRIII and CD16) – necessary for antibody-mediated cellular cytotoxicity (ADCC) (Vivier et al., 2008)– although in the presence of IgG antibodies, these NK cells regain cytotoxic function and specifically lyse human cytomegalovirus (HCMV)-infected cells (Lee et al., 2015).

Despite limited knowledge about metabolic programming in human NK cells, some *in vitro* studies have shown that upregulation of mTOR complex 1 (mTORC1) in CD56^bright^ NK cells, more reflective of a less mature subpopulation, promotes their uptake of glucose and responsiveness to IL-15 signaling (Marçais et al., 2014; Keating et al., 2016; Mao et al., 2016). CD56^bright^ NK-cell proliferation also promotes upregulation of Glut1 and renders these cells metabolically more active than their CD56^dim^ counterparts (Keating et al., 2016). In line with this, glycolysis is necessary for CD56^bright^ NK cells to produce IFN-γ, which is among their primary effector functions in addition to cytotoxicity (Cooper et al., 2001). Therefore, NK cells in tissue may necessitate increased glycolytic activity while maintenance of memory-like populations marked by CD57 and CD16 expression as well as enhanced cytotoxic capacity – akin to memory CD8+ T cells – might require FAO (Kared et al., 2016; Peng and Tian, 2017), although this demands formal testing using *ex vivo* material from healthy subjects as well as in patients with infections (e.g., TB), or malignancies.

## MicroRNAs and Metabolic Programming During Lung Infection Pathology

Inflammation drives the biosynthesis of certain microRNAs (miR) – short molecular structures which post-transcriptionally regulate gene expression – that can directly influence the metabolic profile of immune cells during infectious disease pathogenesis (Iannaccone et al., 2014; Jackson et al., 2017; Sabir et al., 2018; Zhou et al., 2018) and cancer (Thorsson et al., 2018). The Let-7 family of microRNAs reduce the ability of B cells to take up glucose and glutamine from their immediate microenvironment due to downregulation of transport channels, which results in loss of IgM production (Jiang et al., 2018). While Let-7 upregulation has been observed in hepatocellular carcinoma in association with disease severity (Shi et al., 2017), a cancer attributed to viral infection and inflammation, Let-7 upregulation has been associated with protective anti-viral (HIV) cellular CD4+ T-cell responses (Swaminathan et al., 2012). Furthermore, the decreased expression of Let-7 in CD8+ T cells – downstream of T-cell receptor (TCR) activation – allows for the acquisition of effector functions (Wells et al., 2017), underlining that metabolic shifts affect different immune-cell subsets in several ways.

Not only immune cells yet also stromal cells or transformed cells are affected by such metabolic shifts. For instance, miR-155 has been implicated in supporting the growth of breast cancer cells by enhancing glucose metabolism (Kim et al., 2018); the same microRNA also promotes the intracellular survival of *M.tb* by inhibiting autophagy in infected myeloid cells (Etna et al., 2018). A previous study showed that miR-155 is upregulated in peripheral blood mononuclear cells (PBMCs) from patients with active pulmonary TB and a mechanistic *in vitro* approach was used to demonstrate that the Foxhead-box protein O3 (FOXO3) transcription factor is targeted by miR-155 to abrogate apoptosis of *M.tb*-infected cells under normoxic conditions (Huang et al., 2015). Interestingly, FOXO3 is known to be involved in regulating mitochondrial respiration during hypoxia and inhibiting reactive oxygen species (ROS) generation (Ferber et al., 2012), which is also characterized by aberrant vascularization in inflamed tissue (Tafani et al., 2016). Considering that tissue hypoxia is associated with several infectious diseases of the lung, i.e., TB, leishmaniasis (Schatz et al., 2018) and legionellosis (Shankar et al., 1981) as well as solid tumors, FOXO3-mediated activity may increase local apoptosis of transformed cells, which has been shown in the context of cancer (Du et al., 2017).

## The Host Microbiome and Productive Immune Responses to Pulmonary Infections

‘Metabolic programming’ in the host – to a great extent modulated by the gut microbiota – takes place in the small intestine and colon - and affects the quality and quantity of immune responses occurring in other parts of the body (Anand and Mande, 2018; Feng et al., 2018). While translocation of gut pathobionts can induce unfavorable immune responses elsewhere in the body (Manfredo Vieira et al., 2018), inflammatory response originating in the gut can equally influence how the host responds to an invading pulmonary pathogen (Mjosberg and Rao, 2018). *Candida albicans*, a yeast species which is a commonly found member of the human gut microbiome, can activate CD4+ T cells which produce IL-17 and possibly traffic to the lung, where they can specifically cross-react with antigens from *Aspergillus fumigatus* (Bacher et al., 2019), the aetiological agent of invasive pulmonary aspergillosis (IPA). These IL-17 responses were very prominent in patients with airways diseases such as chronic obstructive pulmonary disease, asthma (although this is often associated with a Th2 responses, i.e., IL-4, IL-5 production) and cystic fibrosis (Bacher et al., 2019). Importantly, patients with Crohn’s disease, a debilitating form of inflammatory bowel disease, had high frequencies of IL-17-producing, *C. albicans*-specific CD4+ T cells (Bacher et al., 2019). While dysregulation of the microbiota may lead to activation of IL-17-producing cells which can translocate to the lung to cause pathology locally, the same type of IL-17+ T cells may also be protective in early stages of infection, as shown in preclinical mouse models of pulmonary infections (reviewed by Das and Khader in Das and Khader, 2017). This hypothesis, however, requires formal testing in clinically suitable models which closely resemble the disease immunopathology in humans.

However, not only ‘distant’ effects of tissue/organ-associated microbiomes, i.e., the gut-lung axis, affect lung-resident immune cells, yet the local pulmonary microbiome itself maintains tissue integrity and regulates local immune responses (O’Dwyer et al., 2016). In human avian influenza A H7N9 infection, several FA species were shown to be reduced in the peripheral blood associated with impaired patient survival (Sun et al., 2018). This observation was accompanied by histological evidence of severe lung inflammation, supported by molecular analyses showing downregulation of genes associated with lung epithelial barrier integrity (Sun et al., 2018) – in which the lung microbiome plays a role. Resonating with this observation is the finding that tissue-resident CD8+ T cells require an external supply of FA for their survival, via the activity of fatty-acid-binding proteins 4 and 5 (FABP4 and FABP5) (Pan et al., 2017). This may – at least in part – explain as to why high numbers of activated CD8+ T cells, including those that can home to tissue compartments, are elevated in patients with HIV receiving ART (Mudd and Lederman, 2014), leading to increased circulating cholesterol and triglycerides (Ngala and Fianko, 2013).

Bacteria also produce their own metabolites which can alter immune functions (Feng et al., 2018). FA production by commensal intestinal bacteria – that constitute the host gut microbiome – may complement the FA source needed for memory CD8+ T-cell homeostasis (Luu et al., 2018). Antibiotic-associated clearance of commensal bacterial species can potentially have a drastic effect on the survival of memory CD8+ T cells in the host (Keselman et al., 2016), as shown in mice and warranting confirmation in humans. Microbial metabolites, i.e., short-chain fatty acids (SCFA; acetate, propionate, butyrate) can induce IL-18 production by epithelial cells while also tuning the differentiation of B cells in to plasma cells, antibody production and IgA class switching (Shibata et al., 2017). Butyrate promotes IL-10 production, tight junction formation between epithelia and downregulation of pro-inflammatory responses in human intestinal cells (Zheng et al., 2017). In a murine model of azoxymethane/dextran sodium sulfate (AOM/DSS)-induced colitis, mice treated with a mix of SCFAs (acetate, butyrate, and propionate) were shown to be protected against colon inflammation concomitant with reduced expression of IL-6, TNF-α, and IL-17A (Tian et al., 2018), suggesting regulation of both pro- and anti-inflammatory immune responses by SCFAs. This is in line with clinical observations of patients with inflammatory bowel disease (IBD) such as Crohn’s disease or ulcerative colitis who present with gut microbiota dysbiosis marked by reduced numbers of SCFA-producing bacteria (Goncalves et al., 2018).

Another study in patients with HIV and bacterial pneumonia showed that the indigenous lung microbiome, containing a combination of bacterial communities (annotated by the authors as microbial communities states or MCS) represented by *Pseudomonadaceae* with a mixture of *Sphingomonadaceae* and *Prevotellaceae* (MCS1), was associated with a survival benefit as compared to individual grouped into other MCS characterized by an abundance of *Streptococcaceae* (MCS2A) or *Prevotellaceae* (MCS2B) (Shenoy et al., 2017). Interestingly, patients harboring the MCS1 lung microbiome profile also showed an increase in serum lipid metabolites further to upregulation of T-cell immunoglobulin and mucin domain 3 (TIM-3) in the lungs, based on chromatographic and mRNA analysis, respectively (Shenoy et al., 2017). Using fecal samples provided by individuals from Bangladesh and Tanzania, an intriguing study by Devoto et al. (2019) recently described the discovery and characterization of large bacteriophages containing more than 540 kilobases of genomic material (termed ‘Lak megaphages’) which, based on CRISPR sequences, specifically target *Prevotella* species in the human gut. The authors also found an abundance of *Prevotella* and Lak megaphages in human fecal samples to be inversely correlated. Thus, Lak megaphages may be an important indicator of gut microbiome health, given the benefit provided by *Prevotella* species in health and disease, which may be addressed in future clinical studies using appropriate cohorts.

Several studies in patients with TB have linked the gut microbiome with TB disease activity (reviewed in Hong et al., 2018). Individuals with LTBI exhibited a higher abundance of *Bifidobacterium* spp. in the gut compared to patients with active TB who underwent standard antimycobacterial therapy; higher abundance of *Bacteroides* spp. was found in the gut of patients who were cured of TB following therapy (Wipperman et al., 2017). In this regard, *Bifidobacterium* spp. have been associated with superior immune responses to respiratory infections, while *Bacteroides* spp. have been shown to rather augment antigen-specific, cancer-directed T-cell responses in the host (Forsythe, 2014; Pitt et al., 2017). However, the presence of fat tissue affects the host immune response at different levels: white/beige adipose tissue deposits have been shown to accommodate memory T cells which can provide long-term protection against infections (Han et al., 2017). Conversely, white adipose tissue is also associated with obesity and increased susceptibility to infectious diseases (Hegde and Dhurandhar, 2013), while harboring viral and bacterial pathogens such a HIV and *M.tb* (Damouche et al., 2015; Beigier-Bompadre et al., 2017).

### Microbial Metabolites and Immunomodulation in the Host

Further to the roles of microbial metabolites previously mentioned, SCFAs are able to prime the immune system by increasing the release of IL-18, prompt IgA production by plasma cells and activation of cathelicidin LL-37 (antimicrobial peptide) secretion along with retinoic acid production by APCs to potentiate first-line innate immune responses (Shibata et al., 2017). However, butyrate can also induce Treg development and expansion in a sodium ion-dependent manner, which requires intracellular activation of indolamine-1,2-dioxygenase (IDO) (Gurav et al., 2015), an enzyme necessary for tryptophan metabolism (Taylor and Feng, 1991). Thus, there is a chance that butyrate in combination with IFN-γ – which is the major inducer of IDO expression (Taylor and Feng, 1991) – can promote immune-tolerance in diseased tissue to avoid overt inflammation and also help to fine-tune antigen-specific T-cell responses (Laidlaw et al., 2015). Nevertheless, and dependent on the stage and the nature of infection, Treg engagement may also dampen the formation of productive, pathogen-directed immune responses (Geffner et al., 2009; Lieske et al., 2015). Previous work has shown that depletion of intracellular tryptophan in relation to increasing IDO activation leads to impaired survival of *Chlamydia trachomatis*, which can also be potentiated by IL-1β in combination with low-dose IFN-γ (Carlin and Weller, 1995). Tryptophan is sourced by dietary protein intake by mammals, which in the gut is catabolized by certain species of commensals, i.e., *Bacteroides* sp., *Clostridium* sp., lactobacilli (Roager and Licht, 2018). Metabolites resulting from tryptophan degradation can promote protection against fungal (and potential intracellular bacterial) pathogens in an IL-22- and aryl hydrocarbon receptor (AhR)-dependent mechanism (Zelante et al., 2013). The AhR is generally a toxin-reactive (dioxin) host transcription factor which also has a role in sensing bacterial derivatives to engage immune responses in the lung (Moura-Alves et al., 2014). In mice, AhR has been shown to be indispensable for the development of IL-22+ NKp46+ innate lymphoid cells (ILCs) in the gut (Lee et al., 2011) and the importance of IL-22 in protection against intestinal infections (Guo et al., 2014) as well as lung tissue repair following severe influenza infection (Pociask et al., 2013). Li et al. (2011) described a specialized population of gut-associated AhR-expressing intraepithelial lymphocytes (IELs) which responds to the synthetic tryptophan-derived phytochemical I3C, provides immune surveillance at the epithelial barrier and is able to control bacterial outgrowth following their translocation into the gut lumen. Branched SCFAs, i.e., isovalerate, isobutyrate, and valerate can have an inhibitory effect on histone deacetylases (HDACs), which may in turn downregulate the pro-inflammatory polarization of macrophages in the gut (Chang et al., 2014; Kim, 2018) – based on studies in mice. These findings, therefore, implicate amino acid metabolism, SCFAs and cytokine signaling axes in maintaining host-protective immune mechanisms in tissue during infection and require confirmation in humans.

Long-chain fatty acids (LCFAs) such as arachidonic acid and linoleic acid, both of which are produced by host cells as well as gut bacteria, are known to modulate innate immune responses, i.e., downregulation of IL-6, IL-8, and IL-1β production, increased phagocytic potential of macrophages, increased IL-10 production by epithelial cells and activation of the eicosanoid pathway to produce prostaglandins (Shibata et al., 2017). Arachidonic acid had already been shown (more than 20 years ago) to induce IgE and IL-4 production (strong Th2-skewed immunomodulation) in individuals with atopic dermatitis (Punnonen et al., 1993). Evans et al. (2019) very recently showed that prostaglandin E2 (PGE2) production by the opportunistic fungal pathogen *Cryptococcus neoformans* activates peroxisome proliferator-activated receptor gamma (PPAR-γ) in host macrophages to support intracellular survival and proliferation. Pulmonary *C. neoformans* infections are rare, but can be burdensome and – although treatment with fluconazole is highly effective (Kanjanapradit et al., 2017) – the effect of fluconazole on dysbiosis and immunometabolism is clinically relevant as seen in murine preclinical models (Wheeler et al., 2016) and warrants evaluation in humans.

The exposure of host cells to a broad array of metabolites produced by commensal and invading microbes, as well as the derivates thereof, requires further dissection in humans with different diseases. Future clinical studies may examine, based on previous observations in patients with metabolic disorders (IBD, diabetes, and chronic infections), where biomarkers were available, that metabolite imbalance and subsequent microbiota ‘disruption’ may lead to immune dysfunction (Kim, 2018). Similarly, novel biomarkers in pediatric patients are required to better understand the effect of dysbiosis in early childhood – perpetrated by fatty liver disease, congenital diabetes and drug prescriptions – and whether their value as clinical correlates of susceptibility to infection as well as autoimmune disease (Nash et al., 2017) is relevant or not. Target organs of immune-metabolites may also be distant anatomical sites: The role of the gut-lung axis in accentuating *trans*-anatomical immune responses is being increasingly appreciated (Mjosberg and Rao, 2018; Bacher et al., 2019), providing strong precedence to account for systemic and gastrointestinal health in patients with lung infections with a focus on circulating and locally available metabolites.

### Microbiota and Immune-Modulation by Pre- and Perinatal Nutrition

Metabolic imprinting on immune cells may in fact occur early in life: while a common ante-natal nutritional source in developed nations (at least 80% of mother-child pairs), only a meager 37% of infants under 6 months of age in the developing world (low- to middle-income countries) are breastfed (Victora et al., 2016). Children who do not receive breastmilk in the first few months after birth have been found to be more prone to developing infections and reduced intellectual capabilities (Victora et al., 2016). Furthermore, mothers who do not breastfeed suffer a higher risk of contracting breast or ovarian cancer in their lifetime compared to women who breastfed their babies (Victora et al., 2016). More recently, Moossavi et al. (2019) provided empirical evidence based on a large cohort study of 392 mother-infant dyads (pairs) comprising various ethnicities and found that direct breastfeeding was strongly associated with a higher prevalence of beneficial bacteria, i.e., *Bifidobacterium* supplied to infants as opposed to pumped breastmilk, which had a higher content of potentially pathogenic bacteria such as *Pseudomonas* spp. This would subsequently influence the infant’s metabolic and immune profiles as well as their gut microbiota composition and how they would respond to infections (Le Doare et al., 2018). Breastfeeding has been previously shown to be closely associated with increased protection against bronchiolitis (usually due to a viral infection of the lower respiratory tract) in children under 2 years of age (Wang et al., 2017). Breastmilk composition could, therefore, be a crucial component of future clinical studies investigating immune dynamics as well as treatment outcome following diagnosis of respiratory infections in neo-nates.

All in all, antibiotics directly affect the composition of the microbiota in association with host factors, i.e., the HLA haplotype, lifestyle and environmental factors, i.e., high-fat diets, or uncontrolled glucose intake. These factors may be considered for a more holistic approach in the clinical management of patients with pulmonary infectious diseases. Furthermore, this may not only be conscious decisions of the individual yet also reflect social pressure and socio-economic differences including factors before birth including maternal nutrition, as discussed above.

## Therapeutic Targeting of Host Immunometabolism

Evidence stemming from cancer precision medicine offers a wealth of information to target host immunometabolism for therapeutic purposes, where the tumor microenvironment (TME) in solid tumors is an emerging drug target to induce disease-modifying effects (Le Bourgeois et al., 2018; Vodnala et al., 2019). Much can be learned from the pathophysiological and immunological similarities between the TME and TB lesions in the lung (Vento and Lanzafame, 2011; Bhatt et al., 2012; Dagaonkar et al., 2017), non-tuberculous bacterial pneumonia (Yao et al., 2018) and pulmonary fungal infections (Guimaraes et al., 2013) to be applied for interventional purposes in infectious disease. For instance, the anti-diabetic drug metformin which modulates FAO in memory CD8+ T cells was shown to contribute to improved immune control and better outcomes in a preclinical (murine) TB model (Singhal et al., 2014). In keeping with this observation in mice, a later clinical study demonstrated correlation with desirable clinical outcomes in patients with cancer and T2DM under metformin treatment (Zi et al., 2018). More recently, two retrospective clinical studies showed that metformin use in patients with T2DM, who also contracted pulmonary TB, was associated with improved prognosis after standard anti-TB treatment (Marupuru et al., 2017; Degner et al., 2018) while a third study revealed that patients with T2DM who received metformin therapy exhibited a lower chance of contracting active TB during anti-diabetic treatment follow-up (Lee et al., 2018). Likewise, zileuton, an anti-asthmatic drug [thus representing yet another indication with immunometabolic dysregulation particularly in obese individuals (Periyalil et al., 2013)] which blocks the synthesis of 5-lipoxygenase and thus, leukotriene release, regulates IFN-αβ and IL-1β-associated adverse immunopathology in (murine) TB (Mayer-Barber et al., 2014). Targeting epithelial-cell sphingolipid metabolism using miglustat (marketed as Zavesca^®^, a synthetic analog of D-glucose) resulted in reduced airway inflammation, neutrophilia and *Pseudomonas aeruginosa* burden in a murine infection model (Dechecchi et al., 2011), further strengthening the case for drug-repurposing or multi-purposing.

### Immunotherapy-Based Interventions

Reversing glucose uptake by highly activated effector CD4+ T cells might be a viable option for therapeutic intervention to reduce overt inflammation (Macintyre et al., 2014) and possibly promote CD8+ T-cell activity (Sukumar et al., 2013). However, it may not necessarily affect antigen-specific tissue-resident T-cell subsets, which appear to rely more on lipid metabolism (Pan et al., 2017). Genetic manipulation of T cells, prior to adoptive therapy, may be used to change their metabolic profile and/or susceptibility to certain metabolites by introducing mRNA transcripts of proteins using a newly described microfluidic system, which also measures cell viability and motility in real-time (Jarrell et al., 2019). Analysis of programmed cell-death 1 (PD-1)- and cytotoxic T lymphocyte-associated antigen 4 (CTLA-4)-mediated immune-suppression showed that while the former shifts the cellular metabolic profile from glucose (glycolysis) to lipid-dependence (FAO), CTLA-4 engagement with CD80/86 leads to inhibition of glycolysis without augmenting the requirement for FAO (Patsoukis et al., 2015). Immune checkpoint blockade (ICB) has been particularly successful in patients with metastatic melanoma and lung cancer (Ribas and Wolchok, 2018). PD-1-expressing T cells may, in fact, display a metabolic profile resembling that of memory CD8+ T cells that rely on FAO for survival (Patsoukis et al., 2015). Given that PD-1-mediated therapeutic intervention warrants clinical exploration in infectious diseases (Rao et al., 2017), it is worthwhile bearing in mind that the metabolic reprogramming of pathogen-specific T cells in lung infections is also possible with ICB.

Corrective therapy to improve vascularization in the tumor by inhibiting vascular endothelial growth factor A (VEGF-A) signaling has been clinically beneficial in glioblastoma, lung cancer and age-related macular degeneration (Arjaans et al., 2016). The most widely used anti-VEGF-A monoclonal antibody, bevacizumab, was also shown to rectify aberrant neovascularization and improve small-molecule uptake in TB granulomas in rabbits (Datta et al., 2015). Pharmacological inhibition of angiogenesis with pazopanib (tyrosine kinase inhibitor approved for renal cell cancer) in the zebrafish model of *Mycobacterium marinum* infection – which can recapitulate the early inflammatory events in human pulmonary TB – also enabled improved vascularization and enhanced anti-TB drug penetration into lesions, coupled with reduced bacterial dissemination to other parts of the host (Oehlers et al., 2015). With relevance to immune-cell metabolism, VEGF-A neutralization using bevacizumab in mice fed with a high-fat diet reversed insulin resistance in the liver and adipose tissue, decreasing blood sugar levels (Wu et al., 2014). However, while VEGF-A inhibition improves anti-cancer drug access to the tumor tissue, it has also been shown to reduce tissue oxygenation and induce acute hypoxia in diseased tissue in patients with cancer (Bonekamp et al., 2017; Ueda et al., 2017), unlike the observation within bevacizumab-treated pulmonary TB lesions in rabbits (Datta et al., 2015). VEGF-A utility in chronic infectious disease requires validation in well-designed early phase clinical studies using suitable patient cohorts.

### Immunomodulation by Cholesterol-Lowering Drugs

Statin intake among patients with severe influenza has shown a link with decreased inflammation and MHC class II downregulation in addition to reduction in serum lipids and lower incidence of death (Vandermeer et al., 2012). The use of atorvastatin among patients with HIV infection may result in less activated and less exhausted T-cell populations (downregulation of HLA-DR, TIM-3, and PD-1) while pravastatin contributes to higher numbers of circulating antigen-specific IFN-γ+ CD8+ T cells (Overton et al., 2014). The cellular target of statins is β-hydroxy β-methylglutaryl-CoA (HMG-CoA) reductase (Kwak et al., 2000). A hallmark of statin-based HMG-CoA reductase inhibition is the abrogation of the mevalonate pathway which leads to production of isoprenyl pyrophosphate (IPP) – an intermediary that is indispensable for cholesterol synthesis (Cerqueira et al., 2016). While both atorvastatin (Lipitor^®^) and pravastatin (Pravachol^®^, Selektine^®^) block HMG-CoA reductase aptly, the former does so in a reversible fashion (Holdgate et al., 2003). It also important to mentioned here that, while atorvastatin is more potent than pravastatin at lowering blood cholesterol levels, it also impairs mitochondrial function (Urbano et al., 2017).

In addition to cholesterol synthesis, IPP is also a direct agonist of Vγ9Vδ2 T-cell activation (Wesch et al., 1997). Vγ9Vδ2 T cells represents a particular TCR-γδ population that is prevalent in blood and has been shown to be associated with anti-viral (Agrati et al., 2011; Qin et al., 2011), anti-bacterial (Szereday et al., 2003; Roberts et al., 2007; Qaqish et al., 2017) and anti-tumor (Nakajima et al., 2010; Kobayashi et al., 2011; Nicol et al., 2011) activity. Reduced cholesterol production – due to halted IPP turnover – triggers an upregulation of the low-density lipoprotein (LDL) receptor on cells, leading to an accumulation of intracellular LDL and a dramatic decrease in circulating levels of ‘bad’ cholesterol (Afonso et al., 2018). This phenomenon (high LDLR expression in addition to high intracellular LDL content) in Vγ9Vδ2 T cells has been shown to compromise their immune functions in association with reduced mitochondrial ATP generation and dampened pro-inflammatory functions against breast cancer cells (Rodrigues et al., 2018). Given the growing importance of Vγ9Vδ2 T cells in the host’s armament against infectious agents, i.e., *M.tb*, pathogenic *Escherichia coli*, CMV, *Plasmodium falciparum* etc. (Dechanet et al., 1999; Yoshida, 2001; Wang et al., 2001a,b; Chen et al., 2013; Jagannathan et al., 2014; Schmitt et al., 2017), there is a high likelihood that the use of statins may not promote immune activation but rather immune suppression in patients – most likely dependent on the disease state. Further to Vγ9Vδ2 T cells, inhibition of HMG-CoA also leads to impaired B-cell activation and their subsequent capacity to activate CD4+ T cells via HLA-DR upregulation and antigen presentation (Shimabukuro-Vornhagen et al., 2014). Therefore, statin adjunctive therapy may be considered for inducing anti-inflammatory effect to ameliorate chronic infection rather than to stimulate immune responses for improving clinical outcomes.

Another cholesterol-lowering drug, ezetimibe, has also shown potency against intracellular *M.tb* under hypoxic conditions (Tsai et al., 2017). Furthermore, the study also reported that patients receiving ezetimibe therapy exhibited a lower incidence of LTBI as well as intracellular lipid content, concomitant with reduced bacterial reservoirs (Tsai et al., 2017). However, like statins but via a different mechanism, ezetimibe also has anti-inflammatory properties which downregulates nuclear factor kappa-light-chain-enhancer of activated B cells (NF-κB) expression and monocyte chemotactic protein 1 (MCP-1) production albeit with a chance of increasing NO expression (Toshiyuki and Yasuchika, 2011). NO is a strong mediator of intracellular antimicrobial responses in APCs, i.e., macrophages and dendritic cells, and in modulating T-cell responses (Niedbala et al., 2006; Tripathi et al., 2007). Further to an immunological component, NO is a major intracellular communication molecule involved in vascular integrity and neuronal function (Blaise et al., 2005). Low NO levels was shown to be associated with dyslipidaemia in patients with T2DM, suggesting a role for NO in modulating lipid metabolism (Mishra and Mishra, 2017).

### Non-steroidal Anti-inflammatory Drugs

The role of non-steroidal anti-inflammatory drugs (NSAIDs) such as aspirin and ibuprofen has been previously explored as adjunctive therapy for tissue protection in severe lung infections, including TB (Eisen et al., 2013; Vilaplana et al., 2013; Kroesen et al., 2017) and influenza (Epperly et al., 2016). Although these drugs target cyclooxygenases (COX), crucial enzymes in the eicosanoid pathway for production of lipid mediators such as prostaglandins and leukotrienes, exposure to aspirin in particular has been observed to induce NO synthesis in several cell types (Taubert et al., 2004; Schroder, 2009). Also, aspirin has been shown to induce the expression of nicotinamide adenine dinucleotide phosphate (NADPH) oxidase – which leads to ROS generation – in adipocytes (Vázquez-Meza et al., 2013), that can also harbor *M.tb* bacilli (Beigier-Bompadre et al., 2017). Thus, aspirin-induced NO and ROS production may shift the balance to reflect increased intracellular ROS concentrations without necessarily prompting exaggerated pro-inflammatory cytokine release (Morris et al., 2009; Hsieh and Wang, 2018).

### Sirtuin Activation as an HDT Strategy

Sirtuin 1 (SIRT1) is an enzyme which participates in epigenetic modification of the genome and possesses mono-ADP-ribosyltransferase activity (Kitada and Koya, 2013). The histone deacetylation properties of SIRT1 is involved in regulating the expression of genes involved in lipid and glucose metabolism as well as mitochondrial biogenesis in cells (Kitada and Koya, 2013). In patients with T2DM, SIRT1 expression is downregulated, concomitant with insulin resistance and impaired FAO (Kitada and Koya, 2013). Individuals suffering from obesity showed improvement in lipid metabolism, marked by increased SIRT1 expression, improved mitochondrial turnover and function as well as reduced amounts of intracellular lipid storage and circulating glucose levels (Timmers et al., 2011). Resveratrol-mediated SIRT1 activation has been shown to interrupt the pro-inflammatory activity of CD4+ T cells (Zou et al., 2013), while myeloid cell-specific inactivation of the SIRT1 gene in a murine model of pulmonary TB resulted in amelioration of lung pathology, resolution of chronic inflammation and improved responses to anti-TB drug treatment (Cheng et al., 2017). The therapeutic potential of SIRT1 activation in lung infections – by means of metabolic reprogramming – may suggest more favorable clinical outcomes and warrants the design of appropriate early-phase clinical trials.

### HDT Candidates Currently in Clinical Trials

Cyclic adenosine-monophosphate guanosine monophosphate (cGAMP) is a naturally occurring cyclic dinucleotide structure produced by cyclic GMP-AMP synthase (cGAS) in response to DNA stimulation (Sun et al., 2013). cGAMP also happens to be the natural agonist of the innate immune response-activating molecule stimulator of interferon genes (STING) – which is important for inducing type 1 interferon production (Sun et al., 2013). Administration of exogenous cGAMP followed by STING stimulation was recently shown to result in rectification of glucose and lipid metabolism in mice fed with a high-fat diet by reducing lipid deposition in the liver as well as gluconeogenesis (Guo et al., 2017). Furthermore, cGAMP treatment lead to a decrease in pro-inflammatory responses in hepatocytes and adipocytes concomitant with an increase thereof in myelocytic cells (Guo et al., 2017). A novel STING agonist, ADU-S100, is currently in clinical trials sponsored by Novartis and Aduro Biotech to stimulate anti-cancer immune responses in patients with solid tumors (ClinicalTrials.gov identifiers: NCT02675439, NCT03172936). Preclinical assessment of ADU-S100 showed that, in combination with anti-PD-1 therapy, the drug promotes CD8+ T-cell infiltration into tumors while, on its own, type I interferon and TNF-α induction in haematopoietic cells was observed (Desbien et al., 2018). While immune-stimulation is not necessary for patients with active infection-induced lung pathology, the use of ADU-S100 may be considered to engage innate immune mechanisms as well as tissue-associated T-cell responses (Ohkuri et al., 2017) for eradicating ‘silent’ pathogen reservoirs in individuals harboring asymptomatic/latent infections, i.e., LTBI, *Cryptococcus neoformans* infection in HIV-infected adults (Miller et al., 1996), respiratory viral infections in young children (Chonmaitree et al., 2015) and drug-resistant *Klebsiella pneumoniae* as well as *Acinetobacter baumannii* infections (Weintrob et al., 2013; Qureshi et al., 2014). Considering that cGAMP and STING activation also influences the normalization of metabolic dysregulation in the host, individuals with dyslipidaemia or DM and latent lung infections may also benefit from cGAMP/STING-targeted therapy for enhancing immune responses while correcting their metabolic profile.

Another clinical drug candidate targeting host metabolism is dactolisib (BEZ235), which is currently in early-phase clinical trials for patients with solid cancers (National Library of Medicine, 2018). Dactolisib is an inhibitor of the PI3K/mTOR pathway and was tested in influenza-infected human cells, resulting in strong impairment of glycolysis and a stark reduction in viral titres albeit not affecting virus entry, genome replication and gene transcription in cells (Smallwood et al., 2017). *In vivo* evaluation of the drug (in mice infected with influenza A virus) showed that the drug was well tolerated and a reduction in pathogen burden as well as extension of survival was achieved (Smallwood et al., 2017). This places dactolisib on the list of candidates for host-directed adjunct therapies further to conventional antimicrobials, warranting further evaluation in clinically relevant models representing suitable indications.

**FIGURE 1:**
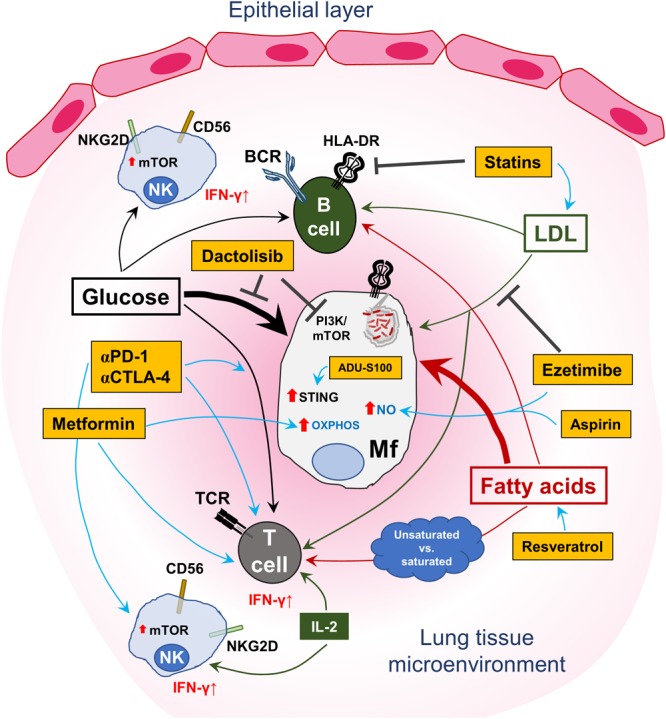
Targeting the host immunometabolism to treat lung infections. A schematic representation of the central role played by glucose and lipid metabolism in immune-cell homeostasis and control of pulmonary infection(s). Uptake of free fatty acids as well as glucose by T and B cells is important for immunological effector functions and maintenance of immune-cell memory. In this regard, saturated fatty acids may have a higher likelihood of promoting anti-inflammatory activity in T cells, while unsaturated fatty acids may lead to the contrary. Antigen-presenting cells (APCs), encompassing macrophages, dendritic cells and B cells, are affected by the intake and endogenous production of fatty acids in their capacity to generate phagolysosomes and upregulate HLA-DR on the cell surface to activate T cells. Similarly, glucose metabolism in B cells is important for cellular proliferation and antibody production. However, infected myeloid cells, represented here by an *M.tb*-infected macrophage (Mf), can disrupt the fatty acid-metabolic balance by increasing consumption of both nutrient types as indicated by the thicker arrows. This loss of equilibrium results in bacterial proliferation, subdued immune activation/modulation and survival of the pathogen in the lung. Furthermore, high levels of circulating low-density lipoprotein (LDL) are also taken up by infected host cells to support intracellular survival of the pathogen. Interspersed in this intricate immuno-metabolic circuit are NK cells, which can also acquire adaptive, memory-like functions and contribute to effective host immune control of pulmonary pathogens. Glucose uptake is also necessary for effector NK cells with regard to IFN-γ production, which is essential for protection against intracellular pathogens. This is concomitant with mTOR upregulation and responsiveness to IL-2. However, the regulation of lipid immunometabolism in NK cells requires further investigation. Also shown in the figure are several drugs (in yellow/orange boxes), most of which are clinically approved except for ADU-S100 and dactolisib, which may be used for targeting the immunometabolic axis in lung infections. Metformin, via the activation of AMPK, can induce oxidative phosphorylation (OXPHOS) in macrophages and improve memory CD8+ T-cell responses (IFN-γ production). Statins block intracellular HMG-CoA reductase and induce an increase in LDL accumulation in exposed cells by upregulating surface expression of the LDL receptor, which can affect both T and B cells by reducing inflammatory responses. Conversely, ezetimibe, which also regulates cholesterol homeostasis, does so by blocking uptake of exogenous LDL. Ezetimibe, like statins, shows a rather anti-inflammatory effect and can induce nitric oxide (NO) production. Aspirin (acetylsalicylic acid), which was already proposed as a possible anti-inflammatory HDT for TB, may also induce NO expression in cells – which is crucial for killing intracellular pathogens. Resveratrol can improve the uptake of free fatty acids by T cells by activating host sirtuin1 (SIRT1) – to fine-tune cellular immune responses while reducing the occurrence of adverse tissue pathology. Although not directly shown, anti-PD-1 and anti-CTLA-4 therapy have been shown to improve glucose metabolism in T-cell populations, in part adding to their clinical anti-tumor activity and may also apply to TB. The investigational clinical drug candidate ADU-S100 mimics cyclic guanosine-monophosphate-adenosine-monophosphate (cGAMP) and can activate the stimulator of interferon genes (STING) protein, thus qualifying it as an immunomodulatory drug candidate with effects on immunometabolism. Dactolisib is currently in early-phase clinical trials to treat patients with solid cancers and has been shown to be beneficial in a preclinical murine influenza infection model – by targeting glycolysis and may apply to TB and staphylococcal infections, where increased glycolysis in host cells supports pathogen growth.

Table 1 is a summary of clinically approved drugs or candidates with HDT properties that can modulate host immune metabolism to improve disease outcomes, which are presently in clinical trials. Figure 1 summarizes the dysregulation of host immunometabolism in pulmonary infections and presents several of the afore-mentioned therapeutic agents that may be considered in a host-directed fashion to restore a dysregulated immunometabolism or to fine-tune the metabolic milieu to the effect of decreasing detrimental immunopathology while enhancing specific and protective immune responses.

**Table 1:** Drugs (clinically approved and candidates in clinical trials) capable of altering host immunometabolism to improve clinical outcomes in pulmonary infections.

Drug/chemical compound	Biological target	Description	References
**Clinically approved agents**
Metformin	Activation of AMPK	Clinically approved to treat T2DM. Increases mitochondrial respiration and FA breakdown, leading to increased generation of memory CD8+ T cells. Shown to enhance immune clearance of *M.tb* in murine models.	Singhal et al., 2014
Bevacizumab	VEGF	Used in the treatment of glioblastoma. Corrects aberrant neovascularization in cancer tissue, allowing for oxygenation and reduction of hypoxia. Has been shown to improve vascular remodeling in TB granulomas, also increasing drug penetration. This is bound to affect immune-cell infiltration and anti-pathogen activity *in situ* due to an increase in oxygen, which enables aerobic glycolysis.	Datta et al., 2015; Oehlers et al., 2015
Ipilimumab	CTLA-4	Both anti-CTLA-4 and anti-PD-1 are clinically approved for treating metastatic melanoma while the latter is also approved for treatment-refractory non-small cell lung cancer. Shown in the cancer setting to cause a shift to FAO from glucose metabolism, reminiscent of memory CD8+ T cells, including reduced uptake of glucose from the extracellular environment, thereby modulating the ability of T cells to acquire effector functions and produce IFN-γ. CTLA-4, on the other hand, inhibits glycolysis without switching the cell metabolism to FAO. This might have implications in patients with diabetes and lung infections, where high blood glucose level is a characteristic; PD-1-expressing antigen-specific T cells may be long-lived (like central memory cells) and highly amenable to therapeutic intervention.	Patsoukis et al., 2015
Nivolumab, pembrolizumab	PD-1		
Statins, i.e., atorvastatin, pravastatin, lovastatin, simvastatin	HMG-CoA reductase	Blocks the enzymatic activity of HMG-CoA reductase which catalyzes an important intermediate step in the isoprenoid pathway: conversion of HMG-CoA to mevalonate. Downstream of this process is the synthesis of isoprenyl pyrophosphate, which is necessary for cholesterol synthesis as well as Vγ9Vδ2 T-cell activation. Statin use in the cancer setting has shown to reduce Vγ9Vδ2 T cell-mediated tumor rejection owing to increased LDLR expression, increased LDL uptake and compromised mitochondrial function. However, statins could be useful against chronic inflammatory processes during infectious disease pathogenesis, i.e., TB.	Wesch et al., 1997; Urbano et al., 2017; Rodrigues et al., 2018
Ezetimibe	Nieman-Pick-C1-Like1 (NPC1L1) protein	Ezetimibe blocks the reabsorption of cholesterol by cells, thereby reducing the amount of intracellular LDL levels. Has anti-inflammatory properties but may induce NO expression. It has been shown to reduce intracellular *M.tb* survival in macrophages while patients with T2DM taking ezetimibe have lower incidence of LTBI.	Toshiyuki and Yasuchika, 2011; Tsai et al., 2017
Aspirin (potentially also other non-steroidal anti-inflammatory drugs, NSAIDs)	Activation of NO/ROS release	NO is an important biological mediator as well as immune effector molecule, particularly against intracellular pathogens – as is the ROS hydrogen peroxide (H_2_O_2_). Maybe involved in lipid metabolism, based on observations in patients with T2DM. Aspirin-driven NO production in macrophages and dendritic cells (as well as adipocytes) may, in fact, promote eradication of local bacterial reservoirs in the case of TB without raising an exaggerated immune response.	Taubert et al., 2004; Blaise et al., 2005; Niedbala et al., 2006; Tripathi et al., 2007; Morris et al., 2009; Schroder, 2009; Eisen et al., 2013; Vázquez-Meza et al., 2013; Vilaplana et al., 2013; Epperly et al., 2016; Beigier-Bompadre et al., 2017; Kroesen et al., 2017; Mishra and Mishra, 2017
Resveratrol (also metformin)	Activation of SIRT1	Sirtiun 1 (SIRT1) is an important histone deacetylase with functions in modulating lipid metabolism as well as immune regulation in myelocytic and lymphocytic cells. Treatment of obese individuals with resveratrol improved lipid metabolism and reduced circulating levels of fatty acids and glucose as well as inflammatory markers. Resveratrol-mediated SIRT1 activation results in dampened pro-inflammatory CD4+ T cells responses as well as resolution of chronic lung inflammation and associated tissue pathology in mice infected with *M.tb*.	Timmers et al., 2011; Zou et al., 2013
Candidates in clinical trials			
ADU-S100	Activation of STING pathway	ADU-S100 is a synthetic cyclic dinucleotide mimicking the structure of cGAMP and is currently in clinical trials as an agonist of the STING pathway. Recent evidence demonstrates that STING activation via cGAMP allows for correction of lipid/glucose metabolism dysregulation while enhancing innate and adaptive immune responses, i.e., type I interferon production and CD8+ T-cell activity. This has implications for eradicating latent pathogen reservoirs, i.e., LTBI, *Cryptococcus* sp. infections, asymptomatic *Klebsiella* spp. infection in individuals, including those who suffer from metabolic conditions/diseases.	(Miller et al., 1996; Weintrob et al., 2013; Qureshi et al., 2014; Chonmaitree et al., 2015; Ohkuri et al., 2017; Desbien et al., 2018); ClinicalTrials.gov identifiers: NCT02675439, NCT03172936
Dactolisib (BEZ235)	PI3K/mTOR pathway	Dactolisib inhibits the PI3K/mTOR pathway to the effect of abrogating glycolysis in exposed cells. This has shown benefit in ameliorating deleterious lung pathology in influenza A infection while extending survival (murine model). Dactolisib is currently in clinical trials for patients with cancer.	Smallwood et al., 2017; National Library of Medicine, 2018

*AMPK, adenosine monophosphate-activated protein kinase; T2DM, type II diabetes mellitus; VEGF, vascular endothelial growth factor; CTLA-4, cytotoxic T lymphocyte-associated antigen 4; PD-1, programmed cell death 1; FAO, fatty acid oxidation; LDL, low-density lipoprotein; NO, nitric oxide; ROS, reactive oxygen species; NSAID, non-steroidal anti-inflammatory drug; cGAMP, cyclic guanosine-monophosphate adenosine-monophosphate; STING, stimulator of interferon genes; LTBI, latent tuberculosis infection; PI3K, phosphatidylinositol-3-kinase; mTOR, mammalian target of rapamycin*.

## Conclusion

The role of cellular metabolism in immunomodulation is an integral component of HDTs. Existing evidence, based on preclinical and clinical observations, suggest that metabolism-targeting drugs given in an adjunct fashion to standard antimicrobial therapy may lead to clinical benefits. In addition, immunometabolic modulatory axes – marked by transcriptional regulation and post-transcriptional as well as epigenetic checkpoints – need to be considered in the process of designing HDTs. An integrated view of information supplied by ongoing clinical trials (‘drug repurposing’) in various patient populations – including dietary practices, genetic backgrounds and exposure to pathogens – is critical in our collective attempts to improve clinical outcomes in treating pulmonary infections.

## Search Criteria

We searched Google, PubMed, and NCBI using the terms ‘immunometabolism,’ ‘immune-metabolism,’ ‘metabolism of immune cells,’ ‘metabolic programming of immune cells,’ and ‘immunometabolic programming’ in the context of and linking pulmonary infections, tuberculosis, diabetes and metabolic diseases. We also used these terms to obtain information concerning relevant drugs and host-directed therapies. All searches for the first submission and revision were performed between June 2018 and March 2019. Irrelevant search results not covered in the scope of this review were eliminated prior to use in discussion for the text.

## Author Contributions

MR wrote the first draft, conceptualized and reviewed all available data and clinical trials. MM conceptualized, developed, wrote, and finalized the review. ED conceptualized and wrote parts of the review. AZ conceptualized and wrote the review.

## Conflict of Interest Statement

The authors declare that the research was conducted in the absence of any commercial or financial relationships that could be construed as a potential conflict of interest.

## References

[B1] Afonso M. S. Machado R. M. Lavrador M. S. Quintao E. C. R. Moore K. J. Lottenberg A. M. (2018). Molecular pathways underlying cholesterol homeostasis. *Nutrients* 10:E760. 10.3390/nu10060760 29899250 PMC6024674

[B2] Agrati C. D’Offizi G. Gougeon M. L. Malkovsky M. Sacchi A. Casetti R. (2011). Innate gamma/delta T-cells during HIV infection: terra relatively incognita in novel vaccination strategies? *AIDS Rev.* 13 3–12. 21412385

[B3] Anand S. Mande S. S. (2018). Diet, microbiota and gut-lung connection. *Front. Microbiol.* 9:2147. 10.3389/fmicb.2018.02147 30283410 PMC6156521

[B4] Arjaans M. Schröder C. P. Oosting S. F. Dafni U. Kleibeuker J. E. de Vries E. G. (2016). VEGF pathway targeting agents, vessel normalization and tumor drug uptake: from bench to bedside. *Oncotarget* 7 21247–21258. 10.18632/oncotarget.6918 26789111 PMC5008282

[B5] Babiker A. Andersson O. Lindblom D. van der Linden J. Wiklund B. Lutjohann D. (1999). Elimination of cholesterol as cholestenoic acid in human lung by sterol 27-hydroxylase: evidence that most of this steroid in the circulation is of pulmonary origin. *J. Lipid Res.* 40 1417–1425. 10428977

[B6] Bacher P. Hohnstein T. Beerbaum E. Röcker M. Blango M. G. Kaufmann S. (2019). Human anti-fungal Th17 immunity and pathology rely on cross-reactivity against *Candida albicans*. *Cell* 176:1340–1355.e15. 10.1016/j.cell.2019.01.041 30799037

[B7] Bagaitkar J. Demuth D. R. Scott D. A. (2008). Tobacco use increases susceptibility to bacterial infection. *Tob. Induc. Dis.* 4:12. 10.1186/1617-9625-4-12 PMC2628337 19094204

[B8] Baldan A. Gomes A. V. Ping P. Edwards P. A. (2008). Loss of ABCG1 results in chronic pulmonary inflammation. *J. Immunol.* 180 3560–3568. 18292583 10.4049/jimmunol.180.5.3560

[B9] Barna B. P. McPeek M. Malur A. Fessler M. B. Wingard C. J. Dobbs L. (2016). Elevated MicroRNA-33 in sarcoidosis and a carbon nanotube model of chronic granulomatous disease. *Am. J. Respir. Cell Mol. Biol.* 54 865–871. 26641802 10.1165/rcmb.2015-0332OC PMC4942222

[B10] Barrero C. A. Datta P. K. Sen S. Deshmane S. Amini S. Khalili K. (2013). HIV-1 Vpr modulates macrophage metabolic pathways: a SILAC-based quantitative analysis. *PLoS One* 8:e68376. 10.1371/journal.pone.0068376 23874603 PMC3709966

[B11] Beigier-Bompadre M. Montagna G. N. Kuhl A. A. Lozza L. Weiner J. I. I. I. Kupz A. (2017). *Mycobacterium tuberculosis* infection modulates adipose tissue biology. *PLoS Pathog.* 13:e1006676. 10.1371/journal.ppat.1006676 29040326 PMC5695609

[B12] Benz C. Reusch U. Muranyi W. Brune W. Atalay R. Hengel H. (2001). Efficient downregulation of major histocompatibility complex class I molecules in human epithelial cells infected with cytomegalovirus. *J. Gen. Virol.* 82 2061–2070. 11514715 10.1099/0022-1317-82-9-2061

[B13] Bhatt M. Kant S. Bhaskar R. (2012). Pulmonary tuberculosis as differential diagnosis of lung cancer. *South Asian J. Cancer* 1 36–42. 10.4103/2278-330X.96507 24455507 PMC3876596

[B14] Bischoff M. Wonnenberg B. Nippe N. Nyffenegger-Jann N. J. Voss M. Beisswenger C. (2017). CcpA affects infectivity of *Staphylococcus aureus* in a hyperglycemic environment. *Front. Cell. Infect. Microbiol.* 7:172. 10.3389/fcimb.2017.00172 28536677 PMC5422431

[B15] Blaise G. A. Gauvin D. Gangal M. Authier S. (2005). Nitric oxide, cell signaling and cell death. *Toxicology* 208 177–192. 15691583 10.1016/j.tox.2004.11.032

[B16] Bonekamp D. Mouridsen K. Radbruch A. Kurz F. T. Eidel O. Wick A. (2017). Assessment of tumor oxygenation and its impact on treatment response in bevacizumab-treated recurrent glioblastoma. *J. Cereb. Blood Flow Metab.* 37 485–494. 10.1177/0271678X16630322. 26861817 PMC5381446

[B17] Borie R. Danel C. Debray M.-P. Taille C. Dombret M.-C. Aubier M. (2011). Pulmonary alveolar proteinosis. *Eur. Respir. Rev.* 20 98–107. 10.1183/09059180.00001311 21632797 PMC9487789

[B18] Bouillon M. El Fakhry Y. Girouard J. Khalil H. Thibodeau J. Mourad W. (2003). Lipid raft-dependent and -independent signaling through HLA-DR molecules. *J. Biol. Chem.* 278 7099–7107. 12499388 10.1074/jbc.M211566200

[B19] Buck M. D. Sowell R. T. Kaech S. M. Pearce E. L. (2017). Metabolic instruction of immunity. *Cell* 169 570–586. 28475890 10.1016/j.cell.2017.04.004 PMC5648021

[B20] Carlin J. M. Weller J. B. (1995). Potentiation of interferon-mediated inhibition of chlamydia infection by interleukin-1 in human macrophage cultures. *Infect. Immun.* 63 1870–1875. 7537250 10.1128/iai.63.5.1870-1875.1995 PMC173237

[B21] Caro-Maldonado A. Wang R. Nichols A. G. Kuraoka M. Milasta S. Sun L. D. (2014). Metabolic reprogramming is required for antibody production that is suppressed in anergic but exaggerated in chronically BAFF-exposed B cells. *J. Immunol.* 192 3626–3636. 10.4049/jimmunol.1302062 24616478 PMC3984038

[B22] Cascone T. McKenzie J. A. Mbofung R. M. Punt S. Wang Z. Xu C. (2018). Increased tumor glycolysis characterizes immune resistance to adoptive T cell therapy. *Cell Metab.* 27 977–987e4. 10.1016/j.cmet.2018.02.024 29628419 PMC5932208

[B23] Casqueiro J. Casqueiro J. Alves C. (2012). Infections in patients with diabetes mellitus: a review of pathogenesis. *Indian J. Endocrinol. Metab.* 16(Suppl. 1) S27–S36. 10.4103/2230-8210.94253 22701840 PMC3354930

[B24] Cerqueira N. M. Oliveira E. F. Gesto D. S. Santos-Martins D. Moreira C. Moorthy H. N. (2016). Cholesterol biosynthesis: a mechanistic overview. *Biochemistry* 55 5483–5506. 27604037 10.1021/acs.biochem.6b00342

[B25] Chang P. V. Hao L. Offermanns S. Medzhitov R. (2014). The microbial metabolite butyrate regulates intestinal macrophage function via histone deacetylase inhibition. *Proc. Natl. Acad. Sci. U.S.A.* 111 2247–2252. 10.1073/pnas.1322269111 24390544 PMC3926023

[B26] Chen C. Y. Yao S. Huang D. Wei H. Sicard H. Zeng G. (2013). Phosphoantigen/IL2 expansion and differentiation of Vgamma2Vdelta2 T cells increase resistance to tuberculosis in nonhuman primates. *PLoS Pathog.* 9:e1003501. 10.1371/journal.ppat.1003501 23966854 PMC3744401

[B27] Cheng C. Y. Gutierrez N. M. Marzuki M. B. Lu X. Foreman T. W. Paleja B. (2017). Host sirtuin 1 regulates mycobacterial immunopathogenesis and represents a therapeutic target against tuberculosis. *Sci. Immunol.* 2:eaaj1789. 10.1126/sciimmunol.aaj1789 28707004 PMC5505666

[B28] Chonmaitree T. Alvarez-Fernandez P. Jennings K. Trujillo R. Marom T. Loeffelholz M. J. (2015). Symptomatic and asymptomatic respiratory viral infections in the first year of life: association with acute otitis media development. *Clin. Infect. Dis.* 60 1–9. 10.1093/cid/ciu714 25205769 PMC4318943

[B29] Cooper M. A. Fehniger T. A. Caligiuri M. A. (2001). The biology of human natural killer-cell subsets. *Trends Immunol.* 22 633–640. 11698225 10.1016/s1471-4906(01)02060-9

[B30] Crawford A. Angelosanto J. M. Nadwodny K. L. Blackburn S. D. Wherry E. J. (2011). A role for the chemokine RANTES in regulating CD8 T cell responses during chronic viral infection. *PLoS Pathog.* 7:e1002098. 10.1371/journal.ppat.1002098 21814510 PMC3141034

[B31] Cury-Boaventura M. F. Gorjao R. de Lima T. M. Newsholme P. Curi R. (2006). Comparative toxicity of oleic and linoleic acid on human lymphocytes. *Life Sci.* 78 1448–1456. 16236329 10.1016/j.lfs.2005.07.038

[B32] Cury-Boaventura M. F. Pompeia C. Curi R. (2004). Comparative toxicity of oleic acid and linoleic acid on jurkat cells. *Clin. Nutr.* 23 721–732. 15297111 10.1016/j.clnu.2003.12.004

[B33] Dagaonkar R. S. Choong C. V. Asmat A. B. Ahmed D. B. A. Chopra A. Lim A. Y. H. (2017). Significance of coexistent granulomatous inflammation and lung cancer. *J. Clin. Pathol.* 70 337–341. 10.1136/jclinpath-2016-203868 27646525 PMC5484024

[B34] Damouche A. Lazure T. Avettand-Fènoël V. Huot N. Dejucq-Rainsford N. Satie A.-P. (2015). Adipose tissue is a neglected viral reservoir and an inflammatory site during chronic HIV and SIV infection. *PLoS Pathog.* 11:e1005153. 10.1371/journal.ppat.1005153 26402858 PMC4581628

[B35] Datta M. Via L. E. Kamoun W. S. Liu C. Chen W. Seano G. (2015). Anti-vascular endothelial growth factor treatment normalizes tuberculosis granuloma vasculature and improves small molecule delivery. *Proc. Natl. Acad. Sci. U.S.A.* 112 1827–1832. 10.1073/pnas.1424563112 25624495 PMC4330784

[B36] Das S. Khader S. (2017). Yin and yang of interleukin-17 in host immunity to infection. *F1000Res.* 6:741. 10.12688/f1000research.10862.1 28713557 PMC5490359

[B37] de Aguiar Vallim T. Q. Lee E. Merriott D. J. Goulbourne C. N. Cheng J. Cheng A. (2017). ABCG1 regulates pulmonary surfactant metabolism in mice and men. *J. Lipid Res.* 58 941–954. 10.1194/jlr.M075101 28264879 PMC5408613

[B38] de Jong A. J. Kloppenburg M. Toes R. E. M. Ioan-Facsinay A. (2014). Fatty acids, lipid mediators, and t-cell function. *Front. Immunol.* 5:483. 10.3389/fimmu.2014.00483 25352844 PMC4195378

[B39] Dechanet J. Merville P. Berge F. Bone-Mane G. Taupin J. L. Michel P. (1999). Major expansion of gammadelta T lymphocytes following cytomegalovirus infection in kidney allograft recipients. *J. Infect. Dis.* 179 1–8. 9841815 10.1086/314568

[B40] Dechecchi M. C. Nicolis E. Mazzi P. Cioffi F. Bezzerri V. Lampronti I. (2011). Modulators of sphingolipid metabolism reduce lung inflammation. *Am. J. Respir. Cell Mol. Biol.* 45 825–833. 10.1165/rcmb.2010-0457OC 21659660

[B41] Degner N. R. Wang J.-Y. Golub J. E. Karakousis P. C. (2018). Metformin use reverses the increased mortality associated with diabetes mellitus during tuberculosis treatment. *Clin. Infect. Dis.* 66 198–205. 10.1093/cid/cix819 29325084 PMC5848303

[B42] Deng C. Xiang Y. Tan T. Ren Z. Cao C. Liu B. (2017). The imbalance of B-lymphocyte subsets in subjects with different glucose tolerance: relationship with metabolic parameter and disease status. *J. Diabetes Res.* 2017:5052812. 10.1155/2017/5052812 28491871 PMC5410374

[B43] Deng T. Lyon C. J. Minze L. J. Lin J. Zou J. Liu J. Z. (2013). Class II major histocompatibility complex plays an essential role in obesity-induced adipose inflammation. *Cell Metab.* 17 411–422. 10.1016/j.cmet.2013.02.009 23473035 PMC3619392

[B44] Desbien A. L. Gauthier K. S. Corrales L. Reiner G. Glickman L. H. Katibah G. (2018). Abstract 631: intratumoral activation of STING with a synthetic cyclic dinucleotide elicits antitumor CD8 T-cell immunity that effectively combines with checkpoint inhibitors. *Cancer Res.* 78:631.

[B45] Devoto A. E. Santini J. M. Olm M. R. Anantharaman K. Munk P. Tung J. (2019). Megaphages infect prevotella and variants are widespread in gut microbiomes. *Nat. Microbiol.* 4 693–700. 10.1038/s41564-018-0338-9 30692672 PMC6784885

[B46] Doughty C. A. Bleiman B. F. Wagner D. J. Dufort F. J. Mataraza J. M. Roberts M. F. (2006). Antigen receptor–mediated changes in glucose metabolism in B lymphocytes: role of phosphatidylinositol 3-kinase signaling in the glycolytic control of growth. *Blood* 107 4458–4465. 16449529 10.1182/blood-2005-12-4788 PMC1895797

[B47] Draper D. W. Madenspacher J. H. Dixon D. King D. H. Remaley A. T. Fessler M. B. (2010). ATP-binding cassette transporter G1 deficiency dysregulates host defense in the lung. *Am. J. Respir. Crit. Care Med.* 182 404–412. 10.1164/rccm.200910-1580OC 20395559 PMC2921600

[B48] Du W. W. Fang L. Yang W. Wu N. Awan F. M. Yang Z. (2017). Induction of tumor apoptosis through a circular RNA enhancing Foxo3 activity. *Cell Death Differ.* 24 357–370. 10.1038/cdd.2016.133 27886165 PMC5299715

[B49] Duque-Correa M. A. Kuhl A. A. Rodriguez P. C. Zedler U. Schommer-Leitner S. Rao M. (2014). Macrophage arginase-1 controls bacterial growth and pathology in hypoxic tuberculosis granulomas. *Proc. Natl. Acad. Sci. U.S.A.* 111 E4024–E4032. 10.1073/pnas.1408839111 25201986 PMC4183271

[B50] Eisen D. P. McBryde E. S. Walduck A. (2013). Low-dose aspirin and ibuprofen’s sterilizing effects on *Mycobacterium tuberculosis* suggest safe new adjuvant therapies for tuberculosis. *J. Infect. Dis.* 208 1925–1927. 10.1093/infdis/jit476 23997233

[B51] Eleftheriadis T. Sounidaki M. Pissas G. Antoniadi G. Liakopoulos V. Stefanidis I. (2016). In human alloreactive CD4(+) T-cells, dichloroacetate inhibits aerobic glycolysis, induces apoptosis and favors differentiation towards the regulatory T-cell subset instead of effector T-cell subsets. *Mol. Med. Rep.* 13 3370–3376. 10.3892/mmr.2016.4912 26935268

[B52] Epperly H. Vaughn F. L. Mosholder A. D. Maloney E. M. Rubinson L. (2016). Nonsteroidal anti-inflammatory drug and aspirin use, and mortality among critically Ill pandemic H1N1 influenza patients: an exploratory analysis. *Jpn. J. Infect. Dis.* 69 248–251. 10.7883/yoken.JJID.2014.577 26255728

[B53] Etna M. P. Sinigaglia A. Grassi A. Giacomini E. Romagnoli A. Pardini M. (2018). *Mycobacterium tuberculosis*-induced miR-155 subverts autophagy by targeting ATG3 in human dendritic cells. *PLoS Pathog.* 14:e1006790. 10.1371/journal.ppat.1006790 29300789 PMC5771628

[B54] Evans R. J. Pline K. Loynes C. A. Needs S. Aldrovandi M. Tiefenbach J. (2019). 15-keto-prostaglandin E2 activates host peroxisome proliferator-activated receptor gamma (PPAR-γ) to promote *Cryptococcus neoformans* growth during infection. *PLoS Pathog.* 15:e1007597. 10.1371/journal.ppat.1007597 30921435 PMC6438442

[B55] Faurholt-Jepsen D. Range N. PrayGod G. Jeremiah K. Faurholt-Jepsen M. Aabye M. G. (2013). Diabetes is a strong predictor of mortality during tuberculosis treatment: a prospective cohort study among tuberculosis patients from Mwanza, Tanzania. *Trop. Med. Int. Health* 18 822–829. 10.1111/tmi.12120 23648145

[B56] Feng Q. Chen W.-D. Wang Y.-D. (2018). Gut microbiota: an integral moderator in health and disease. *Front. Microbiol.* 9:151. 10.3389/fmicb.2018.00151 29515527 PMC5826318

[B57] Ferber E. C. Peck B. Delpuech O. Bell G. P. East P. Schulze A. (2012). FOXO3a regulates reactive oxygen metabolism by inhibiting mitochondrial gene expression. *Cell Death Differ.* 19 968–979. 10.1038/cdd.2011.179 22139133 PMC3354049

[B58] Ferrara G. Murray M. Winthrop K. Centis R. Sotgiu G. Migliori G. B. (2012). Risk factors associated with pulmonary tuberculosis: smoking, diabetes and anti-TNFalpha drugs. *Curr. Opin. Pulm. Med.* 18 233–240. 10.1097/MCP.0b013e328351f9d6 22388583

[B59] Ferrara G. Valentini D. Rao M. Wahlstrom J. Grunewald J. Larsson L. O. (2017). Humoral immune profiling of mycobacterial antigen recognition in sarcoidosis and lofgren’s syndrome using high-content peptide microarrays. *Int. J. Infect. Dis.* 56 167–175. 10.1016/j.ijid.2017.01.021 28159576

[B60] Finlay D. K. Rosenzweig E. Sinclair L. V. Feijoo-Carnero C. Hukelmann J. L. Rolf J. (2012). PDK1 regulation of mTOR and hypoxia-inducible factor 1 integrate metabolism and migration of CD8+ T cells. *J. Exp. Med.* 209 2441–2453. 10.1084/jem.20112607 23183047 PMC3526360

[B61] Fischer K. Hoffmann P. Voelkl S. Meidenbauer N. Ammer J. Edinger M. (2007). Inhibitory effect of tumor cell–derived lactic acid on human T cells. *Blood* 109 3812–3819. 17255361 10.1182/blood-2006-07-035972

[B62] Folco E. J. Sheikine Y. Rocha V. Z. Christen T. Shvartz E. Sukhova G. K. (2011). Hypoxia but not inflammation augments glucose uptake in human macrophages: implications for imaging atherosclerosis with 18fluorine-labeled 2-deoxy-D-glucose positron emission tomography. *J. Am. Coll. Cardiol.* 58 603–614. 10.1016/j.jacc.2011.03.044 21798423

[B63] Forsythe P. (2014). Probiotics and lung immune responses. *Ann. Am. Thorac. Soc.* 11 S33–S37. 10.1513/AnnalsATS.201306-156MG 24437403

[B64] Fox G. J. Lee R. S. Lucas M. Khan F. A. Proulx J.-F. Hornby K. (2015). Inadequate diet is associated with acquiring *Mycobacterium tuberculosis* infection in an inuit community. A case–control study. *Ann. Am. Thorac. Soc.* 12 1153–1162. 10.1513/AnnalsATS.201503-156OC 26099015

[B65] Francois B. Jeannet R. Daix T. Walton A. H. Shotwell M. S. Unsinger J. (2018). Interleukin-7 restores lymphocytes in septic shock: the IRIS-7 randomized clinical trial. *JCI Insight* 3:98960. 10.1172/jci.insight.98960 29515037 PMC5922293

[B66] Freemerman A. J. Johnson A. R. Sacks G. N. Milner J. J. Kirk E. L. Troester M. A. (2014). Metabolic reprogramming of macrophages: glucose transporter 1 (GLUT1)-mediated glucose metabolism drives a proinflammatory phenotype. *J. Biol. Chem.* 289 7884–7896. 10.1074/jbc.M113.522037 24492615 PMC3953299

[B67] Freyberg Z. Harvill E. T. (2017). Pathogen manipulation of host metabolism: a common strategy for immune evasion. *PLoS Pathog.* 13:e1006669. 10.1371/journal.ppat.1006669 29216326 PMC5720515

[B68] Fu Y. Maianu L. Melbert B. R. Garvey W. T. (2004). Facilitative glucose transporter gene expression in human lymphocytes, monocytes, and macrophages: a role for GLUT isoforms 1, 3, and 5 in the immune response and foam cell formation. *Blood Cells Mol. Dis.* 32 182–190. 14757434 10.1016/j.bcmd.2003.09.002

[B69] Garand M. Goodier M. Owolabi O. Donkor S. Kampmann B. Sutherland J. S. (2018). Functional and phenotypic changes of natural killer cells in whole blood during *Mycobacterium tuberculosis* infection and disease. *Front. Immunol.* 9:257. 10.3389/fimmu.2018.00257 29520269 PMC5827559

[B70] Gardiner C. M. (2019). NK cell metabolism. *J. Leukoc. Biol.* 10.1002/JLB.MR0718-260R [Epub ahead of print]. 30676653

[B71] Gardiner C. M. Finlay D. K. (2017). What fuels natural killers? metabolism and NK cell responses. *Front. Immunol.* 8:367. 10.3389/fimmu.2017.00367 28421073 PMC5376555

[B72] Geadas C. Acuna-Villaorduna C. Mercier G. Kleinman M. B. Horsburgh CR Jr Ellner J. J. (2018). FDG-PET/CT activity leads to the diagnosis of unsuspected TB: a retrospective study. *BMC Res. Notes* 11:464. 10.1186/s13104-018-3564-6 30001743 PMC6044021

[B73] Geffner L. Yokobori N. Basile J. Schierloh P. Balboa L. Romero M. M. (2009). Patients with multidrug-resistant tuberculosis display impaired Th1 responses and enhanced regulatory T-cell levels in response to an outbreak of multidrug-resistant *Mycobacterium tuberculosis* M and Ra strains. *Infect. Immun.* 77 5025–5034. 10.1128/IAI.00224-09 19720756 PMC2772532

[B74] Geiger R. Rieckmann J. C. Wolf T. Basso C. Feng Y. Fuhrer T. (2016). L-arginine modulates T cell metabolism and enhances survival and anti-tumor activity. *Cell* 167 829–842.e13. 10.1016/j.cell.2016.09.031 27745970 PMC5075284

[B75] Girardi E. Sane Schepisi M. Goletti D. Bates M. Mwaba P. Yeboah-Manu D. (2017). The global dynamics of diabetes and tuberculosis: the impact of migration and policy implications. *Int. J. Infect. Dis.* 56 45–53. 10.1016/j.ijid.2017.01.018 28153793

[B76] Glasser J. R. Mallampalli R. K. (2012). Surfactant and its role in the pathobiology of pulmonary infection. *Microbes Infect.* 14 17–25. 10.1016/j.micinf.2011.08.019 21945366 PMC3247641

[B77] Gleeson L. E. Sheedy F. J. Palsson-McDermott E. M. Triglia D. O’Leary S. M. O’Sullivan M. P. (2016). Cutting edge: *Mycobacterium tuberculosis* induces aerobic glycolysis in human alveolar macrophages that is required for control of intracellular bacillary replication. *J. Immunol.* 196 2444–2449. 10.4049/jimmunol.1501612 26873991

[B78] Goldschmidt N. Nusair S. Gural A. Amir G. Izhar U. Laxer U. (2003). Disseminated *Mycobacterium kansasii* infection with pulmonary alveolar proteinosis in a patient with chronic myelogenous leukemia. *Am. J. Hematol.* 74 221–223. 14587059 10.1002/ajh.10410

[B79] Goncalves P. Araujo J. R. Di Santo J. P. (2018). A cross-talk between microbiota-derived short-chain fatty acids and the host mucosal immune system regulates intestinal homeostasis and inflammatory bowel disease. *Inflamm. Bowel Dis.* 24 558–572. 10.1093/ibd/izx029 29462379

[B80] Grist J. T. Jarvis L. B. Georgieva Z. Thompson S. Sandhu H. K. Burling K. (2018). Extracellular lactate: a novel measure of T cell proliferation. *J. Immunol.* 200 1220–1226. 10.4049/jimmunol.1700886 29288205 PMC5776880

[B81] Guerrini V. Prideaux B. Blanc L. Bruiners N. Arrigucci R. Singh S. (2018). Storage lipid studies in tuberculosis reveal that foam cell biogenesis is disease-specific. *PLoS Pathog.* 14:e1007223. 10.1371/journal.ppat.1007223 30161232 PMC6117085

[B82] Guimaraes M. D. Marchiori E. Barco Godoy M. C. (2013). Fungal infection mimicking lung cancer: a potential cause of misdiagnosis. *AJR Am. J. Roentgenol.* 201:W364. 23883259 10.2214/AJR.13.10568

[B83] Guo X. Qiu J. Tu T. Yang X. Deng L. Anders R. A. (2014). Induction of innate lymphoid cell-derived interleukin-22 by the transcription factor STAT3 mediates protection against intestinal infection. *Immunity* 40 25–39. 10.1016/j.immuni.2013.10.021 24412612 PMC3919552

[B84] Guo X. Shu C. Li H. Pei Y. Woo S.-L. Zheng J. (2017). Cyclic GMP-AMP ameliorates diet-induced metabolic dysregulation and regulates proinflammatory responses distinctly from STING activation. *Sci. Rep.* 7:6355. 10.1038/s41598-017-05884-y 28743914 PMC5526935

[B85] Gurav A. Sivaprakasam S. Bhutia Y. D. Boettger T. Singh N. Ganapathy V. (2015). Slc5a8, a Na+-coupled high-affinity transporter for short-chain fatty acids, is a conditional tumour suppressor in colon that protects against colitis and colon cancer under low-fibre dietary conditions. *Biochem. J.* 469 267–278. 10.1042/BJ20150242 25984582 PMC4943859

[B86] Haas R. Smith J. Rocher-Ros V. Nadkarni S. Montero-Melendez T. D’Acquisto F. (2015). Lactate regulates metabolic and pro-inflammatory circuits in control of T cell migration and effector functions. *PLoS Biol.* 13:e1002202. 10.1371/journal.pbio.1002202 26181372 PMC4504715

[B87] Han S. J. Glatman Zaretsky A. Andrade-Oliveira V. Collins N. Dzutsev A. Shaik J. (2017). White adipose tissue is a reservoir for memory T cells and promotes protective memory responses to infection. *Immunity* 47 1154–1168.e6. 10.1016/j.immuni.2017.11.009 29221731 PMC5773068

[B88] Hegde V. Dhurandhar N. V. (2013). Microbes and obesity—interrelationship between infection, adipose tissue and the immune system. *Clin. Microbiol. Infect.* 19 314–320. 23506525 10.1111/1469-0691.12157

[B89] Hermans C. Bernard A. (1999). Lung epithelium-specific proteins: characteristics and potential applications as markers. *Am. J. Respir. Crit. Care Med.* 159 646–678. 9927386 10.1164/ajrccm.159.2.9806064

[B90] Holdgate G. A. Ward W. H. J. McTaggart F. (2003). Molecular mechanism for inhibition of 3-hydroxy-3-methylglutaryl CoA (HMG-CoA) reductase by rosuvastatin. *Biochem. Soc. Trans.* 31 528–531. 12773150 10.1042/bst0310528

[B91] Hong B. Y. Paulson J. N. Stine O. C. Weinstock G. M. Cervantes J. L. (2018). Meta-analysis of the lung microbiota in pulmonary tuberculosis. *Tuberculosis* 109 102–108. 10.1016/j.tube.2018.02.006 29559113

[B92] Hotamisligil G. S. (2017). Foundations of immunometabolism and implications for metabolic health and disease. *Immunity* 47 406–420. 10.1016/j.immuni.2017.08.009 28930657 PMC5627521

[B93] Hsieh C.-C. Wang C.-H. (2018). Aspirin disrupts the crosstalk of angiogenic and inflammatory cytokines between 4t1 breast cancer cells and macrophages. *Med. Inflamm.* 2018:6380643. 10.1155/2018/6380643 30034291 PMC6035832

[B94] Huang J. Jiao J. Xu W. Zhao H. Zhang C. Shi Y. (2015). MiR-155 is upregulated in patients with active tuberculosis and inhibits apoptosis of monocytes by targeting FOXO3. *Mol. Med. Rep.* 12 7102–7108. 10.3892/mmr.2015.4250 26324048

[B95] Iannaccone M. Dorhoi A. Kaufmann S. H. (2014). Host-directed therapy of tuberculosis: what is in it for microRNA?. *Expert Opin. Ther. Targets* 18 491–494. 10.1517/14728222.2014.897696 24641181

[B96] Jackson S. E. Redeker A. Arens R. van Baarle D. van den Berg S. P. H. Benedict C. A. (2017). CMV immune evasion and manipulation of the immune system with aging. *Geroscience* 39 273–291. 10.1007/s11357-017-9986-6 28647908 PMC5505894

[B97] Jagannathan P. Kim C. C. Greenhouse B. Nankya F. Bowen K. Eccles-James I. (2014). Loss and dysfunction of Vdelta2(+) gammadelta T cells are associated with clinical tolerance to malaria. *Sci. Transl. Med.* 6:251ra117. 10.1126/scitranslmed.3009793 PMC4198150 25163477

[B98] Jaisinghani N. Dawa S. Singh K. Nandy A. Menon D. Bhandari P. D. (2018). Necrosis driven triglyceride synthesis primes macrophages for inflammation during Mycobacterium tuberculosis infection. *Front. Immunol.* 9:1490. 10.3389/fimmu.2018.01490 30018616 PMC6037689

[B99] Jarrell J. A. Twite A. A. Lau K. H. W. J. Kashani M. N. Priest C. Nieva J. (2019). Intracellular delivery of mRNA to human primary T cells with microfluidic vortex shedding. *Sci. Rep.* 9:3214. 10.1038/s41598-019-40147-y 30824814 PMC6397276

[B100] Jiang S. Yan W. Wang S. E. Baltimore D. (2018). Let-7 suppresses B cell activation through restricting the availability of necessary nutrients. *Cell Metab.* 27 393–403. 29337138 10.1016/j.cmet.2017.12.007

[B101] Käding N. Kaufhold I. Müller C. Szaszák M. Shima K. Weinmaier T. (2017). Growth of Chlamydia pneumoniae is enhanced in cells with impaired mitochondrial function. *Front. Cell. Infect. Microbiol.* 7:499. 10.3389/fcimb.2017.00499 29259924 PMC5723314

[B102] Kanjanapradit K. Kosjerina Z. Tanomkiat W. Keeratichananont W. Panthuwong S. (2017). Pulmonary cryptococcosis presenting with lung mass: report of 7 cases and review of literature. *Clin. Med. Insights Pathol.* 10.1177/1179555717722962 28814908 PMC5546643

[B103] Kared H. Martelli S. Ng T. P. Pender S. L. Larbi A. (2016). CD57 in human natural killer cells and T-lymphocytes. *Cancer Immunol. Immunother.* 65 441–452. 10.1007/s00262-016-1803-z 26850637 PMC11029668

[B104] Keating S. E. Zaiatz-Bittencourt V. Loftus R. M. Keane C. Brennan K. Finlay D. K. (2016). Metabolic reprogramming supports IFN-γ production by CD56bright NK cells. *J. Immunol.* 196 2552–2560. 10.4049/jimmunol.1501783 26873994

[B105] Keselman A. Li E. Maloney J. Singer S. M. (2016). The microbiota contributes to CD8+ T cell activation and nutrient malabsorption following intestinal infection with Giardia duodenalis. *Infect. Immun.* 84 2853–2860. 10.1128/IAI.00348-16 27456829 PMC5038064

[B106] Kim C. H. (2018). Immune regulation by microbiome metabolites. *Immunology* 154 220–229. 10.1111/imm.12930 29569377 PMC5980225

[B107] Kim S. Lee E. Jung J. Lee J. W. Kim H. J. Kim J. (2018). microRNA-155 positively regulates glucose metabolism via PIK3R1-FOXO3a-cMYC axis in breast cancer. *Oncogene* 37 2982–2991. 10.1038/s41388-018-0124-4 29527004 PMC5978802

[B108] Kim T. H. Lee Y. H. Kim K. H. Lee S. H. Cha J. Y. Shin E. K. (2010). Role of lung apolipoprotein A-I in idiopathic pulmonary fibrosis: antiinflammatory and antifibrotic effect on experimental lung injury and fibrosis. *Am. J. Respir. Crit. Care Med.* 182 633–642. 10.1164/rccm.200905-0659OC 20463180

[B109] Kitada M. Koya D. (2013). SIRT1 in type 2 diabetes: mechanisms and therapeutic potential. *Diabetes Metab. J.* 37 315–325. 10.4093/dmj.2013.37.5.315 24199159 PMC3816131

[B110] Knight M. Braverman J. Asfaha K. Gronert K. Stanley S. (2018). Lipid droplet formation in Mycobacterium tuberculosis infected macrophages requires IFN-γ/HIF-1α signaling and supports host defense. *PLoS Pathog.* 14:e1006874. 10.1371/journal.ppat.1006874 29370315 PMC5800697

[B111] Kobayashi H. Tanaka Y. Yagi J. Minato N. Tanabe K. (2011). Phase I/II study of adoptive transfer of gammadelta T cells in combination with zoledronic acid and IL-2 to patients with advanced renal cell carcinoma. *Cancer Immunol. Immunother.* 60 1075–1084. 10.1007/s00262-011-1021-7 21519826 PMC11029699

[B112] Krishnamoorthy N. Abdulnour R. E. Walker K. H. Engstrom B. D. Levy B. D. (2018). Specialized proresolving mediators in innate and adaptive immune responses in airway diseases. *Physiol. Rev.* 98 1335–1370. 10.1152/physrev.00026.2017 29717929 PMC6168922

[B113] Kroesen V. M. Groschel M. I. Martinson N. Zumla A. Maeurer M. van der Werf T. S. (2017). Non-steroidal anti-inflammatory drugs as host-directed therapy for tuberculosis: a systematic review. *Front. Immunol.* 8:772. 10.3389/fimmu.2017.00772 28713389 PMC5492311

[B114] Kulkarni U. Herrmenau C. Win S. J. Bauer M. Kamradt T. (2018). IL-7 treatment augments and prolongs sepsis-induced expansion of IL-10-producing B lymphocytes and myeloid-derived suppressor cells. *PLoS One* 13:e0192304. 10.1371/journal.pone.0192304 29466409 PMC5821326

[B115] Kwak B. Mulhaupt F. Myit S. Mach F. (2000). Statins as a newly recognized type of immunomodulator. *Nat. Med.* 6 1399–1402. 10.1038/82219 11100127

[B116] Laidlaw B. J. Cui W. Amezquita R. A. Gray S. M. Guan T. Lu Y. (2015). Production of IL-10 by CD4(+) regulatory T cells during the resolution of infection promotes the maturation of memory CD8(+) T cells. *Nat. Immunol.* 16 871–879. 10.1038/ni.3224 26147684 PMC4713030

[B117] Laue T. Wrann C. D. Hoffmann-Castendiek B. Pietsch D. Hübner L. Kielstein H. (2015). Altered NK cell function in obese healthy humans. *Bmc Obes.* 2:1. 10.1186/s40608-014-0033-1 26217516 PMC4511543

[B118] Le Bourgeois T. Strauss L. Aksoylar H.-I. Daneshmandi S. Seth P. Patsoukis N. (2018). Targeting T cell metabolism for improvement of cancer immunotherapy. *Front. Oncol.* 8:237. 10.3389/fonc.2018.00237 30123774 PMC6085483

[B119] Le Doare K. Holder B. Bassett A. Pannaraj P. S. (2018). Mother’s milk: a purposeful contribution to the development of the infant microbiota and immunity. *Front. Immunol.* 9:361. 10.3389/fimmu.2018.00361 29599768 PMC5863526

[B120] Lee J. Zhang T. Hwang I. Kim A. Nitschke L. Kim M. (2015). Epigenetic modification and antibody-dependent expansion of memory-like NK cells in human cytomegalovirus-infected individuals. *Immunity* 42 431–442. 10.1016/j.immuni.2015.02.013 25786175 PMC4537797

[B121] Lee J. S. Cella M. McDonald K. G. Garlanda C. Kennedy G. D. Nukaya M. (2011). AHR drives the development of gut ILC22 cells and postnatal lymphoid tissues via pathways dependent on and independent of Notch. *Nat. Immunol.* 13 144–151. 10.1038/ni.2187 22101730 PMC3468413

[B122] Lee M.-C. Chiang C.-Y. Lee C.-H. Ho C.-M. Chang C.-H. Wang J.-Y. (2018). Metformin use is associated with a low risk of tuberculosis among newly diagnosed diabetes mellitus patients with normal renal function: a nationwide cohort study with validated diagnostic criteria. *PLoS One* 13:e0205807. 10.1371/journal.pone.0205807 30335800 PMC6193668

[B123] Levitskaya J. Coram M. Levitsky V. Imreh S. Steigerwald-Mullen P. M. Klein G. (1995). Inhibition of antigen processing by the internal repeat region of the Epstein-Barr virus nuclear antigen-1. *Nature* 375 685–688. 10.1038/375685a0 7540727

[B124] Levy Y. Lacabaratz C. Weiss L. Viard J. P. Goujard C. Lelievre J. D. (2009). Enhanced T cell recovery in HIV-1-infected adults through IL-7 treatment. *J. Clin. Invest.* 119 997–1007. 10.1172/JCI38052 19287090 PMC2662568

[B125] Li Y. Innocentin S. Withers D. R. Roberts N. A. Gallagher A. R. Grigorieva E. F. (2011). Exogenous stimuli maintain intraepithelial lymphocytes via aryl hydrocarbon receptor activation. *Cell* 147 629–640. 10.1016/j.cell.2011.09.025 21999944

[B126] Lieske N. V. Tonby K. Kvale D. Dyrhol-Riise A. M. Tasken K. (2015). Targeting tuberculosis and HIV infection-specific regulatory T cells with MEK/ERK signaling pathway inhibitors. *PLoS One* 10:e0141903. 10.1371/journal.pone.0141903 26544592 PMC4636186

[B127] Lima T. M. Kanunfre C. C. Pompeia C. Verlengia R. Curi R. (2002). Ranking the toxicity of fatty acids on Jurkat and Raji cells by flow cytometric analysis. *Toxicol. In Vitro* 16 741–747. 10.1016/s0887-2333(02)00095-4 12423658

[B128] Lochner M. Berod L. Sparwasser T. (2015). Fatty acid metabolism in the regulation of T cell function. *Trends Immunol.* 36 81–91. 10.1016/j.it.2014.12.005 25592731

[B129] Lonnroth K. Roglic G. Harries A. D. (2014). Improving tuberculosis prevention and care through addressing the global diabetes epidemic: from evidence to policy and practice. *Lancet Diabetes Endocrinol.* 2 730–739. 10.1016/S2213-8587(14)70109-3 25194886

[B130] Lugade A. A. Bogner P. N. Thatcher T. H. Sime P. J. Phipps R. P. Thanavala Y. (2014). Cigarette smoke exposure exacerbates lung inflammation and compromises immunity to bacterial infection. *J. Immunol.* 192 5226–5235. 10.4049/jimmunol.1302584 24752444 PMC4066560

[B131] Luu M. Weigand K. Wedi F. Breidenbend C. Leister H. Pautz S. (2018). Regulation of the effector function of CD8+ T cells by gut microbiota-derived metabolite butyrate. *Sci. Rep.* 8:14430. 10.1038/s41598-018-32860-x 30258117 PMC6158259

[B132] Macintyre A. N. Gerriets V. A. Nichols A. G. Michalek R. D. Rudolph M. C. Deoliveira D. (2014). The glucose transporter Glut1 is selectively essential for CD4 T cell activation and effector function. *Cell Metab.* 20 61–72. 10.1016/j.cmet.2014.05.004 24930970 PMC4079750

[B133] Maeurer M. J. Trinder P. Hommel G. Walter W. Freitag K. Atkins D. (2000). Interleukin-7 or interleukin-15 enhances survival of Mycobacterium tuberculosis-infected mice. *Infect. Immun.* 68 2962–2970. 10.1128/iai.68.5.2962-2970.2000 10768995 PMC97510

[B134] Malashchenko V. V. Meniailo M. E. Shmarov V. A. Gazatova N. D. Melashchenko O. B. Goncharov A. G. (2018). Direct anti-inflammatory effects of granulocyte colony-stimulating factor (G-CSF) on activation and functional properties of human T cell subpopulations in vitro. *Cell Immunol.* 325 23–32. 10.1016/j.cellimm.2018.01.007 29357983

[B135] Manfredo Vieira S. Hiltensperger M. Kumar V. Zegarra-Ruiz D. Dehner C. Khanl N. (2018). Translocation of a gut pathobiont drives autoimmunity in mice and humans. *Science* 359 1156–1161. 10.1126/science.aar7201 29590047 PMC5959731

[B136] Mao Y. van Hoef V. Zhang X. Wennerberg E. Lorent J. Witt K. (2016). IL-15 activates mTOR and primes stress-activated gene expression leading to prolonged antitumor capacity of NK cells. *Blood* 128 1475–1489. 10.1182/blood-2016-02-698027 27465917 PMC5025899

[B137] Marais B. J. Lonnroth K. Lawn S. D. Migliori G. B. Mwaba P. Glaziou P. (2013). Tuberculosis comorbidity with communicable and non-communicable diseases: integrating health services and control efforts. *Lancet Infect. Dis.* 13 436–448. 10.1016/S1473-3099(13)70015-X 23531392

[B138] Marçais A. Cherfils-Vicini J. Viant C. Degouve S. Viel S. Fenis A. (2014). The metabolic checkpoint kinase mTOR is essential for IL-15 signaling during the development and activation of NK cells. *Nat. Immunol.* 15 749–757. 10.1038/ni.2936 24973821 PMC4110708

[B139] Martínez-González M. Á. Hershey M. S. Zazpe I. Trichopoulou A. (2017). Transferability of the Mediterranean diet to non-Mediterranean countries. what is and what is not the Mediterranean diet. *Nutrients* 9:E1226. 10.3390/nu9111226 PMC5707698 29117146

[B140] Martins A. Han J. Kim S. O. (2010). The multifaceted effects of granulocyte colony-stimulating factor in immunomodulation and potential roles in intestinal immune homeostasis. *Iubmb Life* 62 611–617. 10.1002/iub.361 20681025 PMC2916186

[B141] Marupuru S. Senapati P. Pathadka S. Miraj S. S. Unnikrishnan M. K. Manu M. K. (2017). Protective effect of metformin against tuberculosis infections in diabetic patients: an observational study of south Indian tertiary healthcare facility. *Braz. J. Infect. Dis.* 21 312–316. 10.1016/j.bjid.2017.01.001 28199824 PMC9427654

[B142] Mayer-Barber K. D. Andrade B. B. Oland S. D. Amaral E. P. Barber D. L. Gonzales J. (2014). Host-directed therapy of tuberculosis based on interleukin-1 and type I interferon crosstalk. *Nature* 511 99–103. 10.1038/nature13489 24990750 PMC4809146

[B143] Miller K. D. Mican J. A. M. Davey R. T. (1996). Asymptomatic solitary pulmonary nodules due to Cryptococcus neoformans in patients infected with human immunodeficiency virus. *Clin. Infect. Dis.* 23 810–812. 10.1093/clinids/23.4.810 8909849

[B144] Mishra S. Mishra B. (2017). Study of lipid peroxidation, nitric oxide end product, and trace element status in type 2 diabetes mellitus with and without complications. *Int. J. Appl. Basic Med. Res.* 7 88–93. 10.4103/2229-516X.205813 28584737 PMC5441270

[B145] Mjosberg J. Rao A. (2018). Lung inflammation originating in the gut. *Science* 359 36–37. 10.1126/science.aar4301 29302003

[B146] Moore K. J. Sheedy F. J. Fisher E. A. (2013). Macrophages in atherosclerosis: a dynamic balance. *Nat. Rev. Immunol.* 13 709–721. 10.1038/nri3520 23995626 PMC4357520

[B147] Moossavi S. Sepehri S. Robertson B. Bode L. Goruk S. Field C. J. (2019). Composition and variation of the human milk microbiota are influenced by maternal and early-life factors. *Cell Host Microbe* 25 324–335. 30763539 10.1016/j.chom.2019.01.011

[B148] Morris T. Stables M. Hobbs A. de Souza P. Colville-Nash P. Warner T. Newson J. Bellingan G. (2009). Effects of low-dose aspirin on acute inflammatory responses in humans. *J. Immunol.* 183 2089–2096. 10.4049/jimmunol.0900477 19597002

[B149] Moura-Alves P. Fae K. Houthuys E. Dorhoi A. Kreuchwig A. Furkert J. (2014). AhR sensing of bacterial pigments regulates antibacterial defence. *Nature* 512 387–392. 10.1038/nature13684 25119038

[B150] Mudd J. C. Lederman M. M. (2014). CD8 T cell persistence in treated HIV infection. *Curr. Opin. HIV Aids* 9 500–505. 10.1097/COH.0000000000000086 25010897 PMC4211072

[B151] National Library of Medicine (ed.) (2018). *Dactolisib.* Bethesda, MD: National Library of Medicine.

[B152] Nakajima J. Murakawa T. Fukami T. Goto S. Kaneko T. Yoshida Y. (2010). A phase I study of adoptive immunotherapy for recurrent non-small-cell lung cancer patients with autologous gammadelta T cells. *Eur. J. Cardiothorac. Surg.* 37 1191–1197. 10.1016/j.ejcts.2009.11.051 20137969

[B153] Nash M. J. Frank D. N. Friedman J. E. (2017). Early microbes modify immune system development and metabolic homeostasis—the “Restaurant” hypothesis revisited. *Front. Endocrinol.* 8:349. 10.3389/fendo.2017.00349 29326657 PMC5733336

[B154] Newton R. Priyadharshini B. Turka L. A. (2016). Immunometabolism of regulatory T cells. *Nat. Immunol.* 17 618–625. 10.1038/ni.3466 27196520 PMC5006394

[B155] Ngala R. A. Fianko K. (2013). Dyslipidaemia and dysglycaemia in HIV-infected patients on highly active anti-retroviral therapy in Kumasi Metropolis. *Afr. Health Sci.* 13 1107–1116. 10.4314/ahs.v13i4.35 24940339 PMC4056472

[B156] Nicol A. J. Tokuyama H. Mattarollo S. R. Hagi T. Suzuki K. Yokokawa K. (2011). Clinical evaluation of autologous gamma delta T cell-based immunotherapy for metastatic solid tumours. *Br. J. Cancer* 105 778–786. 10.1038/bjc.2011.293 21847128 PMC3171009

[B157] Niedbala W. Cai B. Liew F. Y. (2006). Role of nitric oxide in the regulation of T cell functions. *Ann. Rheum. Dis.* 65 iii37–iii40. 10.1136/ard.2006.058446 17038470 PMC1798386

[B158] O’Dwyer D. N. Dickson R. P. Moore B. B. (2016). The lung microbiome, immunity and the pathogenesis of chronic lung disease. *J. Immunol.* 196 4839–4847. 10.4049/jimmunol.1600279 27260767 PMC4894335

[B159] Oehlers S. H. Cronan M. R. Scott N. R. Thomas M. I. Okuda K. S. Walton E. M. (2015). Interception of host angiogenic signalling limits mycobacterial growth. *Nature* 517 612–615. 10.1038/nature13967 25470057 PMC4312197

[B160] Ohkuri T. Kosaka A. Ishibashi K. Kumai T. Hirata Y. Ohara K. (2017). Intratumoral administration of cGAMP transiently accumulates potent macrophages for anti-tumor immunity at a mouse tumor site. *Cancer Immunol. Immunother.* 66 705–716. 10.1007/s00262-017-1975-1 28243692 PMC11028681

[B161] O’Shea D. Cawood T. J. O’Farrelly C. Lynch L. (2010). Natural killer cells in obesity: impaired function and increased susceptibility to the effects of cigarette smoke. *PLoS One* 5:e8660. 10.1371/journal.pone.0008660 20107494 PMC2801590

[B162] O’Sullivan D. van der Windt G. J. Huang S. C. Curtis J. D. Chang C. H. Buck M. D. (2014). Memory CD8(+) T cells use cell-intrinsic lipolysis to support the metabolic programming necessary for development. *Immunity* 41 75–88. 10.1016/j.immuni.2014.06.005 25001241 PMC4120664

[B163] Overton E. T. Sterrett S. Westfall A. O. Kahan S. M. Burkholder G. Zajac A. J. (2014). Effects of atorvastatin and pravastatin on immune activation and T-cell function in ART-suppressed HIV-1 infected patients. *Aids* 28 2627–2631. 10.1097/QAD.0000000000000475 25574964 PMC4338916

[B164] Pachón-Peña G. Serena C. Ejarque M. Petriz J. Duran X. Oliva-Olivera W. (2016). Obesity determines the immunophenotypic profile and functional characteristics of human mesenchymal stem cells from adipose tissue. *Stem Cells Transl. Med.* 5 464–475. 10.5966/sctm.2015-0161 26956208 PMC4798735

[B165] Palmer C. S. Anzinger J. J. Zhou J. Gouillou M. Landay A. Jaworowski A. (2014a). Glucose transporter 1–expressing proinflammatory monocytes are elevated in combination antiretroviral therapy–treated and untreated HIV+ subjects. *J. Immunol.* 193 5595–5603. 10.4049/jimmunol.1303092 25367121

[B166] Palmer C. S. Ostrowski M. Gouillou M. Tsai L. Yu D. Zhou J. (2014b). Increased glucose metabolic activity is associated with CD4+ T-cell activation and depletion during chronic HIV infection. *Aids* 28 297–309. 10.1097/QAD.0000000000000128 24335483 PMC4293200

[B167] Pan Y. Tian T. Park C. O. Lofftus S. Y. Mei S. Liu X. (2017). Survival of tissue-resident memory T cells requires exogenous lipid uptake and metabolism. *Nature* 543 252–256. 10.1038/nature21379 28219080 PMC5509051

[B168] Patsoukis N. Bardhan K. Chatterjee P. Sari D. Liu B. Bell L. N. (2015). PD-1 alters T-cell metabolic reprogramming by inhibiting glycolysis and promoting lipolysis and fatty acid oxidation. *Nat. Commun.* 6:6692. 10.1038/ncomms7692 25809635 PMC4389235

[B169] Pearce E. L. Walsh M. C. Cejas P. J. Harms G. M. Shen H. Wang L. S. (2009). Enhancing CD8 T-cell memory by modulating fatty acid metabolism. *Nature* 460 103–107. 10.1038/nature08097 19494812 PMC2803086

[B170] Peng H. Tian Z. (2017). Natural killer cell memory: progress and implications. *Front. Immunol.* 8:1143. 10.3389/fimmu.2017.01143 28955346 PMC5601391

[B171] Perez L. M. Bernal A. de Lucas B. San Martin N. Mastrangelo A. Garcia A. (2015). Altered metabolic and stemness capacity of adipose tissue-derived stem cells from obese mouse and human. *PLoS One* 10:e0123397. 10.1371/journal.pone.0123397 25875023 PMC4395137

[B172] Periyalil H. A. Gibson P. G. Wood L. G. (2013). Immunometabolism in obese asthmatics: are we there yet? *Nutrients* 5 3506–3530. 10.3390/nu5093506 24025484 PMC3798918

[B173] Peyron P. Vaubourgeix J. Poquet Y. Levillain F. Botanch C. Bardou F. (2008). Foamy macrophages from tuberculous patients’ granulomas constitute a nutrient-rich reservoir for M. tuberculosis persistence. *PLoS Pathog.* 4:e1000204. 10.1371/journal.ppat.1000204 19002241 PMC2575403

[B174] Pitt J. M. Vetizou M. Gomperts Boneca I. Lepage P. Chamaillard M. Zitvogel L. (2017). Enhancing the clinical coverage and anticancer efficacy of immune checkpoint blockade through manipulation of the gut microbiota. *Oncoimmunology* 6:e1132137. 10.1080/2162402X.2015.1132137 28197360 PMC5283646

[B175] Pociask D. A. Scheller E. V. Mandalapu S. McHugh K. J. Enelow R. I. Fattman C. L. (2013). IL-22 is essential for lung epithelial repair following influenza infection. *Am. J. Pathol.* 182 1286–1296. 10.1016/j.ajpath.2012.12.007 23490254 PMC3620404

[B176] Prosser G. Brandenburg J. Reiling N. Barry C. E. Wilkinson R. J. Wilkinson K. A. (2017). The bacillary and macrophage response to hypoxia in tuberculosis and the consequences for T cell antigen recognition. *Microbes Infect.* 19 177–192. 10.1016/j.micinf.2016.10.001 27780773 PMC5335906

[B177] Punnonen J. Punnonen K. Jansen C. T. Kalimo K. (1993). Interferon (IFN)-alpha, IFN-gamma, interleukin (IL)-2, and arachidonic acid metabolites modulate IL-4-induced IgE synthesis similarly in healthy persons and in atopic dermatitis patients. *Allergy* 48 189–195. 10.1111/j.1398-9995.1993.tb00712.x 8506987

[B178] Qaqish A. Huang D. Chen C. Y. Zhang Z. Wang R. Li S. (2017). Adoptive transfer of phosphoantigen-specific gammadelta T cell subset attenuates Mycobacterium tuberculosis infection in nonhuman primates. *J. Immunol.* 198 4753–4763. 10.4049/jimmunol.1602019 28526681 PMC5557270

[B179] Qin G. Liu Y. Zheng J. Ng I. H. Y. Xiang Z. Lam K.-T. (2011). Type 1 responses of human Vγ9Vδ2 T cells to influenza A viruses. *J. Virol.* 85 10109–10116. 10.1128/JVI.05341-11 21752902 PMC3196408

[B180] Qureshi Z. A. Syed A. Clarke L. G. Doi Y. Shields R. K. (2014). Epidemiology and clinical outcomes of patients with carbapenem-resistant *klebsiella pneumoniae* bacteriuria. *Antimicrob. Agents Chemother.* 58 3100–3104. 10.1128/AAC.02445-13 24637691 PMC4068487

[B181] Rao M. Ippolito G. Mfinanga S. Ntoumi F. Yeboah-Manu D. Vilaplana C. (2019a). Improving treatment outcomes for MDR-TB - Novel host-directed therapies and personalised medicine of the future. *Int. J. Infect. Dis.* 80S S62–S67. 30685590 10.1016/j.ijid.2019.01.039

[B182] Rao M. Ligeiro D. Maeurer M. (2019b). Precision medicine in the clinical management of respiratory tract infections including multidrug-resistant tuberculosis: learning from innovations in immuno-oncology. *Curr. Opin. Pulm. Med.* 25 233–241. 10.1097/mcp.0000000000000575 30883448

[B183] Rao M. Valentini D. Dodoo E. Zumla A. Maeurer M. (2017). Anti-PD-1/PD-L1 therapy for infectious diseases: learning from the cancer paradigm. *Int. J. Infect. Dis.* 56 221–228. 10.1016/j.ijid.2017.01.028 28163164

[B184] Rao M. Valentini D. Poiret T. Dodoo E. Parida S. Zumla A. (2015). B in TB: B cells as mediators of clinically relevant immune responses in tuberculosis. *Clin. Infect. Dis.* 61(Suppl. 3) S225–S234. 26409285 10.1093/cid/civ614 PMC4583574

[B185] Rao M. Vogelzang A. Kaiser P. Schuerer S. Kaufmann S. H. Gengenbacher M. (2013). The tuberculosis vaccine candidate Bacillus Calmette-Guerin Delta*ureC::hly* coexpressing human interleukin-7 or -18 enhances antigen-specific T cell responses in mice. *PLoS One* 8:e78966. 10.1371/journal.pone.0078966 24236077 PMC3827306

[B186] Remmerie A. Scott C. L. (2018). Macrophages and lipid metabolism. *Cell Immunol.* 330 27–42. 10.1126/science.aar4060 29429624 PMC6108423

[B187] Ribas A. Wolchok J.D. (2018). Cancer immunotherapy using checkpoint blockade. *Science* 359 1350–1355. 29567705 10.1126/science.aar4060 PMC7391259

[B188] Roager H. M. Licht T. R. (2018). Microbial tryptophan catabolites in health and disease. *Nat. Commun.* 9:3294. 30120222 10.1038/s41467-018-05470-4 PMC6098093

[B189] Roberts T. Beyers N. Aguirre A. Walzl G. (2007). Immunosuppression during active tuberculosis is characterized by decreased interferon- gamma production and CD25 expression with elevated forkhead box P3 transforming growth factor- beta, and interleukin-4 mRNA levels. *J. Infect. Dis.* 195 870–878. 10.1086/511277 17299718

[B190] Rodrigues N. V. Correia D. V. Mensurado S. Nobrega-Pereira S. deBarros A. Kyle-Cezar F. (2018). Low Density Lipoprotein uptake inhibits the activation and antitumor functions of human Vgamma9Vdelta2 T cells. *Cancer Immunol. Res.* 6 448–457. 10.1158/2326-6066.cir-17-0327 29358174

[B191] Roy Chowdhury R. Vallania F. Yang Q. Lopez Angel C. J. Darboe F. Penn-Nicholson A. (2018). A multi-cohort study of the immune factors associated with *M. tuberculosis* infection outcomes. *Nature* 560 644–648. 10.1038/s41586-018-0439-x 30135583 PMC6414221

[B192] Rupp J. Gieffers J. Klinger M. Van Zandbergen G. Wrase R. Maass M. (2007). *Chlamydia pneumoniae* directly interferes with HIF-1α stabilization in human host cells. *Cell Microbiol.* 9 2181–2191. 10.1111/j.1462-5822.2007.00948.x 17490410

[B193] Sabir N. Hussain T. Shah S. Z. A. Peramo A. Zhao D. Zhou X. (2018). miRNAs in tuberculosis: new avenues for diagnosis and host-directed therapy. *Front. Microbiol.* 9:602. 10.3389/fmicb.2018.00602 29651283 PMC5885483

[B194] Schatz V. Neubert P. Rieger F. Jantsch J. (2018). Hypoxia, hypoxia-inducible factor-1α, and innate antileishmanial immune responses. *Front. Immunol.* 9:216. 10.3389/fimmu.2018.00216 29520262 PMC5827161

[B195] Schlums H. Cichocki F. Tesi B. Theorell J. Beziat V. Tim D. (2015). Cytomegalovirus infection drives adaptive epigenetic diversification of NK cells with altered signaling and effector function. *Immunity* 42 443–456. 10.1016/j.immuni.2015.02.008 25786176 PMC4612277

[B196] Schmitt M. Schmitt A. Wiesneth M. Huckelhoven A. Wu Z. Kuball J. (2017). Peptide vaccination in the presence of adjuvants in patients after hematopoietic stem cell transplantation with CD4+ T cell reconstitution elicits consistent CD8+ T cell responses. *Theranostics* 7 1705–1718. 10.7150/thno.18301 28529646 PMC5436522

[B197] Schroder H. (2009). Nitric oxide and aspirin: a new mediator for an old drug. *Am. J. Ther.* 16 17–23. 10.1097/mjt.0b013e318164bd60 19092646

[B198] Schweitzer S. C. Reding A. M. Patton H. M. Sullivan T. P. Stubbs C. E. Villalobos-Menuey E. (2006). Endogenous versus exogenous fatty acid availability affects lysosomal acidity and MHC class II expression. *J. Lipid Res.* 47 2525–2537. 10.1194/jlr.m600329-jlr200 16914769

[B199] Sen S. Kaminiski R. Deshmane S. Langford D. Khalili K. Amini S. (2015). Role of Hexokinase-1 in the survival of HIV-1-infected macrophages. *Cell Cycle* 14 980–989. 10.1080/15384101.2015.1006971 25602755 PMC4612415

[B200] Sethumadhavan S. Silva M. Philbrook P. Nguyen T. Hatfield S. M. Ohta A. (2017). Hypoxia and hypoxia-inducible factor (HIF) downregulate antigen-presenting MHC class I molecules limiting tumor cell recognition by T cells. *PLoS One* 12:e0187314. 10.1371/journal.pone.0187314 29155844 PMC5695785

[B201] Shah B. R. Hux J. E. (2003). Quantifying the risk of infectious diseases for people with diabetes. *Diabetes Care* 26 510–513. 10.2337/diacare.26.2.510 12547890

[B202] Shaikh S. R. Mitchell D. Carroll E. Li M. Schneck J. Edidin M. (2008). Differential effects of a saturated and a monounsaturated fatty acid on MHC class I antigen presentation. *Scand. J. Immunol.* 68 30–42. 10.1111/j.1365-3083.2008.02113.x 18533931 PMC2805012

[B203] Shankar P. S. Anderson C. L. Scott J. H. (1981). Legionnaires’ disease with severe hypoxemia and saddleback fever. *Postgrad. Med.* 69 87–92. 7010336 10.1080/00325481.1981.11715730

[B204] Shehata H. M. Murphy A. J. Lee M. K. S. Gardiner C. M. Crowe S. M. Sanjabi S. (2017). Sugar or Fat?-metabolic requirements for immunity to viral infections. *Front. Immunol.* 8:1311. 10.3389/fimmu.2017.01311 29085369 PMC5649203

[B205] Shenoy M. K. Iwai S. Lin D. L. Worodria W. Ayakaka I. Byanyima P. (2017). Immune response and mortality risk relate to distinct lung microbiomes in patients with HIV and pneumonia. *Am. J. Respir. Crit. Care Med.* 195 104–114. 10.1164/rccm.201603-0523oc 27447987 PMC5214918

[B206] Shi L. Eugenin E. A. Subbian S. (2016). Immunometabolism in tuberculosis. *Front. Immunol.* 7:150. 10.3389/fimmu.2016.00150 27148269 PMC4838633

[B207] Shi W. Zhang Z. Yang B. Guo H. Jing L. Liu T. (2017). Overexpression of microRNA let-7 correlates with disease progression and poor prognosis in hepatocellular carcinoma. *Medicine* 96:e7764. 10.1097/md.0000000000007764 28796071 PMC5556237

[B208] Shibata N. Kunisawa J. Kiyono H. (2017). Dietary and microbial metabolites in the regulation of host immunity. *Front. Microbiol.* 8:2171 10.3389/fmicb.2017.02171 PMC5681998 29163449

[B209] Shimabukuro-Vornhagen A. Zoghi S. Liebig T. M. Wennhold K. Chemitz J. Draube A. (2014). Inhibition of protein geranylgeranylation specifically interferes with CD40-dependent B cell activation, resulting in a reduced capacity to induce T cell immunity. *J. Immunol.* 193 5294–5305. 10.4049/jimmunol.1203436 25311809

[B210] Singh V. Kaur C. Chaudhary V. K. Rao K. V. Chatterjee S. (2015). *M. tuberculosis* secretory protein ESAT-6 induces metabolic flux perturbations to drive foamy macrophage differentiation. *Sci. Rep.* 5:12906. 26250836 10.1038/srep12906 PMC5388048

[B211] Singhal A. Jie L. Kumar P. Hong G. S. Leow M. K. Paleja B. (2014). Metformin as adjunct antituberculosis therapy. *Sci. Transl. Med.* 6:263ra159. 10.1126/scitranslmed.3009885 25411472

[B212] Smallwood H. S. Duan S. Morfouace M. Rezinciuc S. Shulkin B. L. Shelat A. (2017). Targeting metabolic reprogramming by influenza infection for therapeutic intervention. *Cell Rep.* 19 1640–1653. 10.1016/j.celrep.2017.04.039 28538182 PMC5599215

[B213] Sportes C. Hakim F. T. Memon S. A. Zhang H. Chua K. S. Brown M. R. (2008). Administration of rhIL-7 in humans increases in vivo TCR repertoire diversity by preferential expansion of naive T cell subsets. *J. Exp. Med.* 205 1701–1714. 10.1084/jem.20071681 18573906 PMC2442646

[B214] Sreejit G. Ahmed A. Parveen N. Jha V. Valluri V. L. Ghosh S. (2014). The ESAT-6 protein of *Mycobacterium tuberculosis* interacts with beta-2-microglobulin (beta2M) affecting antigen presentation function of macrophage. *PLoS Pathog.* 10:e1004446. 10.1371/journal.ppat.1004446 25356553 PMC4214792

[B215] Subbian S. Tsenova L. Kim M. J. Wainwright H. C. Visser A. Bandyopadhyay N. (2015). Lesion-specific immune response in granulomas of patients with pulmonary tuberculosis: a pilot study. *PLoS One* 10:e0132249. 10.1371/journal.pone.0132249 26133981 PMC4489805

[B216] Sukumar M. Liu J. Ji Y. Subramanian M. Crompton J. G. Yu Z. (2013). Inhibiting glycolytic metabolism enhances CD8+ T cell memory and antitumor function. *J. Clin. Invest.* 123 4479–4488. 10.1172/jci69589 24091329 PMC3784544

[B217] Sun L. Wu J. Du F. Chen X. Chen Z. J. (2013). Cyclic GMP-AMP synthase is a cytosolic DNA sensor that activates the type I interferon pathway. *Science* 339 786–791. 10.1126/science.1232458 23258413 PMC3863629

[B218] Sun X. Song L. Feng S. Li L. Yu H. Wang Q. (2018). Fatty acid metabolism is associated with disease severity after H7N9 infection. *EBioMedicine* 33 218–229. 10.1016/j.ebiom.2018.06.019 29941340 PMC6085509

[B219] Swaminathan S. Suzuki K. Seddiki N. Kaplan W. Cowley M. J. Hood C. L. (2012). Differential regulation of the Let-7 family of microRNAs in CD4+ T cells alters IL-10 expression. *J. Immunol.* 188 6238–6246. 10.4049/jimmunol.1101196 22586040

[B220] Szaszak M. Shima K. Kading N. Hannus M. Solbach W. Rupp J. (2013). Host metabolism promotes growth of *Chlamydia pneumoniae* in a low oxygen environment. *Int. J. Med. Microbiol.* 303 239–246. 10.1016/j.ijmm.2013.03.005 23665044

[B221] Szereday L. Baliko Z. Szekeres-Bartho J. (2003). Gamma/delta T cell subsets in patients with active *Mycobacterium tuberculosis* infection and tuberculin anergy. *Clin. Exp. Immunol.* 131 287–291. 10.1046/j.1365-2249.2003.02063.x 12562390 PMC1808624

[B222] Tafani M. Sansone L. Limana F. Arcangeli T. De Santis E. Polese M. (2016). The interplay of reactive oxygen species, hypoxia, inflammation, and sirtuins in cancer initiation and progression. *Oxid. Med. Cell. Longev.* 2016:3907147. 26798421 10.1155/2016/3907147 PMC4699039

[B223] Taubert D. Berkels R. Grosser N. Schröder H. Gründemann D. Schömig E. (2004). Aspirin induces nitric oxide release from vascular endothelium: a novel mechanism of action. *Br. J. Pharmacol.* 143 159–165. 10.1038/sj.bjp.0705907 15289285 PMC1575268

[B224] Taylor M. W. Feng G. S. (1991). Relationship between interferon-gamma, indoleamine 2,3-dioxygenase, and tryptophan catabolism. *FASEB J.* 5 2516–2522. 10.1096/fasebj.5.11.1907934 1907934

[B225] Theurich S. Tsaousidou E. Hanssen R. Lempradl A. M. Mauer J. Timper K. (2017). IL-6/Stat3-dependent induction of a distinct, obesity-associated NK cell subpopulation deteriorates energy and glucose homeostasis. *Cell Metab.* 26:171-184.e6. 28683285 10.1016/j.cmet.2017.05.018

[B226] Thomassen M. J. Barna B. P. Malur A. G. Bonfield T. L. Farver C. F. Malur A. (2007). ABCG1 is deficient in alveolar macrophages of GM-CSF knockout mice and patients with pulmonary alveolar proteinosis. *J. Lipid Res.* 48 2762–2768. 10.1194/jlr.p700022-jlr200 17848583

[B227] Thorsson V. Gibbs D. L. Brown S. D. Wolf D. Bortone D. S. Ou T. H. (2018). The immune landscape of cancer. *Immunity* 48:812-830.e14. 10.1016/j.immuni.2018.03.023 PMC5982584 29628290

[B228] Tian Y. Xu Q. Sun L. Ye Y. Ji G. (2018). Short-chain fatty acids administration is protective in colitis-associated colorectal cancer development. *J. Nutr. Biochem.* 57 103–109. 10.1016/j.jnutbio.2018.03.007 29694938

[B229] Timmers S. Konings E. Bilet L. Houtkooper R. H. van de Weijer T. Goossens G. H. (2011). Calorie restriction-like effects of 30 days of resveratrol supplementation on energy metabolism and metabolic profile in obese humans. *Cell Metab.* 14 612–622. 10.1016/j.cmet.2011.10.002 22055504 PMC3880862

[B230] Toshiyuki I. Yasuchika T. (2011). Ezetimibe and vascular inflammation. *Curr. Vasc. Pharmacol.* 9 99–108. 10.2174/157016111793744788 21044014

[B231] Tripathi P. Tripathi P. Kashyap L. Singh V. (2007). The role of nitric oxide in inflammatory reactions. *FEMS Immunol. Med. Microbiol.* 51 443–452. 10.1111/j.1574-695x.2007.00329.x 17903207

[B232] Tsai I. F. Kuo C. P. Lin A. B. Chien M. N. Ho H. T. Wei T. Y. (2017). Potential effect of ezetimibe against *Mycobacterium tuberculosis* infection in type II diabetes. *Respirology* 22 559–566. 10.1111/resp.12948 27879023

[B233] Ueda S. Saeki T. Osaki A. Yamane T. Kuji I. (2017). Bevacizumab induces acute hypoxia and cancer progression in patients with refractory breast cancer: multimodal functional imaging and multiplex cytokine analysis. *Clin. Cancer Res.* 23 5769–5778. 10.1158/1078-0432.ccr-17-0874 28679773

[B234] Urbano F. Bugliani M. Filippello A. Scamporrino A. Di Mauro S. Di Pino A. (2017). Atorvastatin but not pravastatin impairs mitochondrial function in human pancreatic islets and rat β-cells. Direct effect of oxidative stress. *Sci. Rep.* 7:11863. 10.1038/s41598-017-11070-x PMC5605712 28928397

[B235] van der Windt G. J. Everts B. Chang C. H. Curtis J. D. Freitas T. C. Amiel E. (2012). Mitochondrial respiratory capacity is a critical regulator of CD8+ T cell memory development. *Immunity* 36 68–78. 10.1016/j.immuni.2011.12.007 22206904 PMC3269311

[B236] van Enschot J. W. van Balkom R. H. (2013). Sarcoidosis following *Mycobacterium tuberculosis* infection: coincidence or consequence. *Respir. Med. Case Rep.* 9 11–14. 10.1016/j.rmcr.2013.03.006 26029621 PMC3949549

[B237] Vandermeer M. L. Thomas A. R. Kamimoto L. Reingold A. Gershman K. Meek J. (2012). Association between use of statins and mortality among patients hospitalized with laboratory-confirmed influenza virus infections: a multistate study. *J. Infect. Dis.* 205 13–19. 10.1093/infdis/jir695 22170954

[B238] Vassena L. Miao H. Cimbro R. Malnati M. S. Cassina G. Proschan M. A. (2012). Treatment with IL-7 prevents the decline of circulating CD4+ T cells during the acute phase of SIV infection in rhesus macaques. *PLoS Pathog.* 8:e1002636. 10.1371/journal.ppat.1002636 22511868 PMC3325214

[B239] Vázquez-Meza H. de Piña M. Z. Pardo J. P. Riveros-Rosas H. Villalobos-Molina R. Piña E. (2013). Non-steroidal anti-inflammatory drugs activate NADPH oxidase in adipocytes and raise the H(2)O(2) pool to prevent cAMP-stimulated protein kinase a activation and inhibit lipolysis. *BMC Biochem.* 14:13. 10.1186/1471-2091-14-13 23718778 PMC3671242

[B240] Vento S. Lanzafame M. (2011). Tuberculosis and cancer: a complex and dangerous liaison. *Lancet Oncol.* 12 520–522. 10.1016/s1470-2045(11)70105-x 21624773

[B241] Victora C. G. Bahl R. Barros A. J. D. França G. V. A. Horton S. Krasevec J. (2016). Breastfeeding in the 21st century: epidemiology, mechanisms, and lifelong effect. *Lancet* 387 475–490. 10.1016/s0140-6736(15)01024-7 26869575

[B242] Vilaplana C. Marzo E. Tapia G. Diaz J. Garcia V. Cardona P. J. (2013). Ibuprofen therapy resulted in significantly decreased tissue bacillary loads and increased survival in a new murine experimental model of active tuberculosis. *J. Infect. Dis.* 208 199–202. 10.1093/infdis/jit152 23564636

[B243] Vitko N. P. Spahich N. A. Richardson A. R. (2015). Glycolytic dependency of high-level nitric oxide resistance and virulence in *Staphylococcus aureus*. *mBio* 6:e00045-15. 25852157 10.1128/mBio.00045-15 PMC4453550

[B244] Vivier E. Tomasello E. Baratin M. Walzer T. Ugolini S. (2008). Functions of natural killer cells. *Nat. Immunol.* 9 503–510. 18425107 10.1038/ni1582

[B245] Vodnala S. K. Eil R. Kishton R. J. Sukumar M. Yamamoto T. N. Ha N.-H. (2019). T cell stemness and dysfunction in tumors are triggered by a common mechanism. *Science* 363:eaau0135. 10.1126/science.aau0135 30923193 PMC8194369

[B246] Wang J. Ramette A. Jurca M. Goutaki M. Beardsmore C. S. Kuehni C. E. (2017). Breastfeeding and respiratory tract infections during the first 2 years of life. *ERJ Open Res.* 3:00143-2016. 10.1183/23120541.00143-2016 PMC5464122 28616408

[B247] Wang J. Y. Lee L. N. Yu C. J. Chien Y. J. Yang P. C. Tami G. (2009). Factors influencing time to smear conversion in patients with smear-positive pulmonary tuberculosis. *Respirology* 14 1012–1019. 10.1111/j.1440-1843.2009.01598.x 19659516

[B248] Wang L. Das H. Kamath A. Bukowski J. F. (2001a). Human V gamma 2V delta 2 T cells produce IFN-gamma and TNF-alpha with an on/off/on cycling pattern in response to live bacterial products. *J. Immunol.* 167 6195–6201. 10.4049/jimmunol.167.11.6195 11714780

[B249] Wang L. Kamath A. Das H. Li L. Bukowski J. F. (2001b). Antibacterial effect of human V gamma 2V delta 2 T cells in vivo. *J. Clin. Invest.* 108 1349–1357. 11696580 10.1172/JCI13584 PMC209444

[B250] Wei J. Raynor J. Nguyen T.-L. M. Chi H. (2017). Nutrient and metabolic sensing in T cell responses. *Front. Immunol.* 8:247. 10.3389/fimmu.2017.00247 28337199 PMC5343023

[B251] Weintrob A. C. Murray C. K. Lloyd B. Li P. Lu D. Miao Z. (2013). Active surveillance for asymptomatic colonization with multidrug-resistant gram-negative bacilli among injured service members – A three-year evaluation. *MSMR* 20 17–22. 24011372 PMC4095982

[B252] Wells A. C. Daniels K. A. Angelou C. C. Fagerberg E. Burnside A. S. Markstein M. (2017). Modulation of let-7 miRNAs controls the differentiation of effector CD8 T cells. *eLife* 6:e26398. 28737488 10.7554/eLife.26398 PMC5550279

[B253] Wesch D. Marx S. Kabelitz D. (1997). Comparative analysis of alpha beta and gamma delta T cell activation by *Mycobacterium tuberculosis* and isopentenyl pyrophosphate. *Eur. J. Immunol.* 27 952–956. 10.1002/eji.1830270422 9130649

[B254] Wheeler M. L. Limon J. J. Bar A. S. Leal C. A. Gargus M. Tang J. (2016). Immunological consequences of intestinal fungal dysbiosis. *Cell Host Microbe* 19 865–873. 10.1016/j.chom.2016.05.003 27237365 PMC4900921

[B255] Wilson A. M. Nair P. Hargreave F. E. Efthimiadis A. E. Anvari M. Allen C. J. (2011). Lipid and smoker’s inclusions in sputum macrophages in patients with airway diseases. *Respir. Med.* 105 1691–1695. 10.1016/j.rmed.2011.07.011 21831624

[B256] Wipperman M. F. Fitzgerald D. W. Juste M. A. J. Taur Y. Namasivayam S. Sher A. (2017). Antibiotic treatment for Tuberculosis induces a profound dysbiosis of the microbiome that persists long after therapy is completed. *Sci. Rep.* 7:10767. 28883399 10.1038/s41598-017-10346-6 PMC5589918

[B257] Wofford J. A. Wieman H. L. Jacobs S. R. Zhao Y. Rathmell J. C. (2008). IL-7 promotes Glut1 trafficking and glucose uptake via STAT5-mediated activation of Akt to support T-cell survival. *Blood* 111 2101–2111. 10.1182/blood-2007-06-096297 18042802 PMC2234050

[B258] Wojcik A. J. Skaflen M. D. Srinivasan S. Hedrick C. C. (2008). A critical role for ABCG1 in macrophage inflammation and lung homeostasis. *J. Immunol.* 180 4273–4282. 10.4049/jimmunol.180.6.4273 18322240

[B259] Wright J. R. (2004). Host defense functions of pulmonary surfactant. *Biol. Neonate* 85 326–332. 10.1159/000078172 15211087

[B260] Wu L. E. Meoli C. C. Mangiafico S. P. Fazakerley D. J. Cogger V. C. Mohamad M. (2014). Systemic VEGF-A neutralization ameliorates diet-induced metabolic dysfunction. *Diabetes Metab. Res. Rev.* 63 2656–2667. 10.2337/db13-1665 24696450

[B261] Yao D. Zhang L. Wu P. L. Gu X. L. Chen Y. F. Wang L. X. (2018). Clinical and misdiagnosed analysis of primary pulmonary lymphoma: a retrospective study. *BMC Cancer* 18:281. 10.1186/s12885-018-4184-1 29530011 PMC5848441

[B262] Yoshida N. (2001). Role of gamma/delta T-cells in the peripheral blood of patients with pulmonary tuberculosis. *Kurume Med. J.* 48 175–181. 10.2739/kurumemedj.48.175 11501500

[B263] Zelante T. Iannitti R. G. Cunha C. De Luca A. Giovannini G. Pieraccini G. (2013). Tryptophan catabolites from microbiota engage aryl hydrocarbon receptor and balance mucosal reactivity via interleukin-22. *Immunity* 39 372–385. 10.1016/j.immuni.2013.08.003 23973224

[B264] Zhang W. Yu H. Shang J. Liu T. Ma J. Gu X. (2015). Association between dietary habits and recurrent respiratory infection in children: a case–control study. *J. Tradit. Chin. Med. Sci.* 2 105–110. 10.1016/j.jtcms.2016.01.003

[B265] Zheng L. Kelly C. J. Battista K. D. Schaefer R. Lanis J. M. Alexeev E. E. (2017). Microbial-derived butyrate promotes epithelial barrier function through il-10 receptor-dependent repression of claudin-2. *J. Immunol.* 199 2976–2984. 10.4049/jimmunol.1700105 28893958 PMC5636678

[B266] Zhou X. Li X. Wu M. (2018). miRNAs reshape immunity and inflammatory responses in bacterial infection. *Signal Transduct. Target. Ther.* 3:14. 29844933 10.1038/s41392-018-0006-9 PMC5968033

[B267] Zi F. Zi H. Li Y. He J. Shi Q. Cai Z. (2018). Metformin and cancer: an existing drug for cancer prevention and therapy. *Oncol. Lett.* 15 683–690. 29422962 10.3892/ol.2017.7412 PMC5772929

[B268] Zou T. Yang Y. Xia F. Huang A. Gao X. Fang D. (2013). Resveratrol inhibits CD4+ T cell activation by enhancing the expression and activity of sirt1. *PLoS One* 8:e75139. 10.1371/journal.pone.0075139 24073240 PMC3779207

[B269] Zumla A. Rao M. Wallis R. S. Kaufmann S. H. Rustomjee R. Mwaba P. (2016). Host-directed therapies for infectious diseases: current status, recent progress, and future prospects. *Lancet Infect. Dis.* 16 e47–e63. 10.1016/S1473-3099(16)00078-5 27036359 PMC7164794

